# Nanoplastic Translocation Across Biological Barriers (Blood–Brain, Placental, Intestinal): Transport Mechanisms, Tissue-Specific Vulnerabilities, and a Corona-Driven Barrier Selectivity Framework

**DOI:** 10.3390/biology15141133

**Published:** 2026-07-12

**Authors:** Ahmet Ali Berber, Esra Yıldız, Nurcan Berber, Muammer Kurnaz, Nihan Akıncı Kenanoğlu

**Affiliations:** 1 Vocational School of Health Services, Çanakkale Onsekiz Mart University, 17100 Canakkale, Türkiye; aberber@comu.edu.tr (A.A.B.); nberber@comu.edu.tr (N.B.); 2 Faculty of Science, Department of Biology, Sakarya University, 54050 Sakarya, Türkiye; 3 Kelkit Sema Doğan Vocational School of Health Services, Gümüşhane Üniversitesi, 29600 Gümüşhane, Türkiye; muammerkurnazz@gmail.com; 4 Faculty of Science, Department of Biology, Çanakkale Onsekiz Mart University, 17100 Canakkale, Türkiye; nakinci@comu.edu.tr

**Keywords:** nanoplastics, microplastics, blood–brain barrier, placental barrier, intestinal barrier, protein corona, receptor-mediated transcytosis, tight junctions, corona-driven barrier selectivity

## Abstract

Nanoplastics (NPs; ≤1 µm) have been detected in human placenta, blood, lung, atherosclerotic plaque, testis, semen, olfactory bulb, and brain, shifting the field from environmental description toward mechanistic interrogation of barrier crossing. This review synthesises current evidence on NP translocation across the intestinal epithelium, the blood–brain barrier (BBB) and the placental syncytiotrophoblast. We distinguish four evidence categories throughout the review: detection, association, mechanism, and causality. We also apply model-system labels (in silico, in vitro, ex vivo, animal, and human) to every mechanistic claim, so that the strength of each statement can be read off directly. Most current studies use pristine polystyrene nanoplastics at concentrations 3–6 orders of magnitude above plausible human exposure, so the mechanistic conclusions below are hypothesis-generating for human disease rather than definitive. We propose a working conceptual framework—corona-driven barrier selectivity (CDBS)—in which the particle–corona–surface complex, rather than the bare polymer, is hypothesised to dictate which receptor and transport machinery (TfR1, LRP1, FcRn, P-gp/BCRP, caveolae) each barrier engages. CDBS is offered as a hypothesis-stage organising tool requiring experimental validation, not as an established mechanism. We outline reported transport modes, including clathrin- and caveolin-mediated endocytosis, paracellular leakage via ROS-induced tight-junction disassembly, receptor-mediated transcytosis, and the candidate olfactory route. We emphasise that pristine polystyrene-bead doses commonly exceed plausible human exposure by 3–6 orders of magnitude, that detection methods underestimate sub-micrometre particles, and that causal links between NPs and human disease remain hypothesis-generating. A research agenda built on weathered reference materials, microphysiological systems, and integrative human biomarker science is proposed.

## Simple Summary

Nanoplastics—plastic fragments smaller than one micrometre—have been detected in human blood, placenta, lung, atherosclerotic plaque, and brain tissue, raising concerns about their ability to cross protective barriers. This review examines how nanoplastics may pass through three critical barriers: the intestinal lining, the blood–brain barrier, and the placenta. We synthesise the cellular routes involved and the tissue-specific vulnerabilities that follow entry. We also propose a working hypothesis—corona-driven barrier selectivity—in which the biomolecular coating of each nanoplastic and the receptors at each barrier jointly determine crossing. Most experimental doses far exceed realistic human exposure, and causal links to human disease remain to be established.

## 1. Introduction

Plastic production exceeds 400 Mt per year, and weathering generates micrometric (microplastics, MPs; 1 µm–5 mm) and submicrometric (nanoplastics, NPs; ≤1 µm) fragments now detected across air, water, soil and biota [1,2]. Although polymer signatures have been reported in a widening range of human samples, several recurrent caveats apply across this literature: (i) detection of a polymer signature is not the same as quantification of intact particles; (ii) reported concentrations vary by 1–3 orders of magnitude between laboratories analysing similar matrices [3,4]; and (iii) procedural blanks, where reported, frequently account for a substantial fraction of the apparent sample signal [5,6].

Within these caveats, polymer signatures have been described in human stool [7], placenta by both vibrational spectroscopy [8] and by pyrolysis-gas-chromatography–mass-spectrometry (Py-GC/MS) [9], lung tissue [10,11], whole blood [12,13], atherosclerotic plaque [14], olfactory bulb [15], testis and semen [16,17], and the frontal cortex of decedent brains [18]. Marfella et al. [14] reported, in a single-centre prospective study of 304 patients undergoing carotid endarterectomy, that detection of MNPs in the excised plaque was associated with a 4.5-fold higher hazard of myocardial infarction, stroke, or all-cause death over 34 months. The strength of this hazard ratio is striking, but the result is from one centre, has not yet been independently replicated, and is subject to potential residual confounding and contamination concerns acknowledged by the authors. It should therefore be regarded as hypothesis-generating evidence of association, not as proof of causality. Table 1 consolidates the human biomonitoring evidence stream across tissues, with analytical method, evidence level, and evidence category for each entry.

Distinguishing methodological artefact from biological signal has become the central interpretive problem in this literature. Three artefact classes recur: (i) contamination from plastic-containing labware, reagents and airborne fibres, which produces false positives at all size ranges and disproportionately at the low-concentration end; (ii) polymer misassignment by automated library-matching against complex biological matrices, particularly for pigment particles and lipid-rich tissues; and (iii) apparent detection of sub-micrometre particles below the true spatial resolution of µ-Raman or µ-FTIR, which biases size distributions upward and can produce spurious quantifications. A biological signal is credible when the polymer identification is orthogonal (for example, Py-GC/MS plus vibrational spectroscopy plus electron microscopy), when procedural blanks are reported and subtracted transparently, and when recovery efficiency is characterised on matrix-matched spiked tissue. Where these controls are incomplete, the finding should be read as a provisional detection rather than as a confirmed biological presence.

Three barriers are of disproportionate clinical and developmental importance and form the focus of this review: the intestinal barrier, the principal portal for ingested NPs and the interface with the gut microbiome; the blood–brain barrier (BBB), whose integrity is essential for neuronal homeostasis; and the placental barrier, where the syncytiotrophoblast governs foetal exposure during the critical window of organogenesis. Each is structurally and molecularly distinct, yet each must contend with the same nanoscale challenger.

### 1.1. A Working Conceptual Framework: Corona-Driven Barrier Selectivity

**Figure 1:**
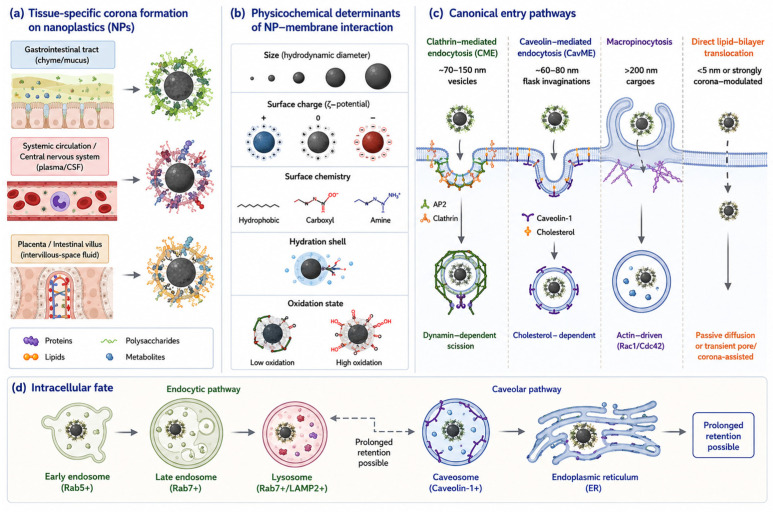
Nanoplastic interaction with cell membranes and cellular entry pathways within the corona-driven barrier selectivity (CDBS) framework. (**a**) Tissue-specific corona formation. On contact with biological fluid, nanoplastics (NPs) acquire compartment-specific biomolecular coronae: polysaccharide- and mucin-enriched in the gastrointestinal tract (green); protein- and lipoprotein-dominated in plasma/CSF (pink); and mixed protein–lipid–metabolite in placental and intestinal interstitial fluids (orange). (**b**) Physicochemical determinants of NP–membrane interaction. Five master variables jointly determine corona affinity and membrane wrapping: hydrodynamic diameter, surface ζ-potential (cationic/neutral/anionic), surface chemistry (hydrophobic vs. carboxyl/amine groups), hydration shell, and oxidation state acquired during weathering. (**c**) Canonical entry pathways. Clathrin-mediated endocytosis (CME; ~70–150 nm cargoes, AP2/clathrin coat, dynamin scission); caveolin-mediated endocytosis (CavME; ~60–80 nm cholesterol- and caveolin-1-rich invaginations); macropinocytosis (>200 nm cargoes, actin-driven, Rac1/Cdc42-regulated); and direct lipid-bilayer translocation (<5 nm or strongly corona-modulated). (**d**) Intracellular fate. The endocytic pathway (Rab5^+^ early endosome → Rab7^+^ late endosome → Rab7^+^/LAMP2^+^ lysosome) supports prolonged lysosomal retention, whereas the caveolar pathway (caveolin-1^+^ caveosome → endoplasmic reticulum) bypasses lysosomal degradation and is the principal candidate route for transcytosis. The CDBS framework hypothesises that compartment-specific coronae bias which entry pathway is engaged and therefore which intracellular fate predominates at each barrier. Abbreviations: AP2, adaptor protein 2; CSF, cerebrospinal fluid; LAMP2, lysosomal-associated membrane protein 2. Arrows indicate the direction of nanoparticle transport or intracellular trafficking. Different colors are used to distinguish biological pathways and cellular compartments for visual clarity only and do not indicate quantitative differences.

To organise the comparative discussion that follows, we use a working conceptual framework that we refer to as corona-driven barrier selectivity (CDBS). The framework rests on three premises with independent literature support: (i) within milliseconds of contact with biological fluid, NPs acquire a tissue- and fluid-specific protein and lipid corona [19,20]; (ii) the corona, rather than the bare polymer, presents the molecular surface that engages cell-surface receptors and influences membrane interaction [21,22]; and (iii) each barrier expresses a distinct receptor and transport repertoire [23,24,25]. Taken together, these premises predict that the same NP species may translocate the intestinal, BBB, and placental interfaces with different efficiencies and through different routes, because its corona is sampled differently by each fluid compartment and engaged differently by the available receptor surfaces (Figure 1). The integrated framework itself has not been experimentally validated, and we present it as a hypothesis-stage organising tool rather than an established mechanism (further discussed in Section 1.3). Table 2 maps compartment-specific corona partners to candidate receptor and transport machinery at each barrier and provides an evidence-level tag for each pairing.

### 1.2. Aims and Approach

We pursue three mechanistic questions, applied comparatively across the three barriers: (i) what is known mechanistically about the trajectory of NPs from extracellular fluid to the abluminal compartment; (ii) what is contradictory between in silico, in vitro, ex vivo, animal, and human findings; and (iii) what is missing—which mechanistic claims are unsupported, which controls are systematically absent, and which experimental frameworks would best resolve the field. Throughout, we apply explicit evidence-level labels to mechanistic claims (in silico/in vitro monoculture/in vitro co-culture/animal in vivo/ex vivo human/human in vivo), and we hedge claims for which only one or two studies, frequently in the same model system, are available; Table 3 summarises the principal mechanistic findings for each barrier together with their explicit evidence levels.

### 1.3. Distinguishing CDBS from Existing Corona and Transcytosis Models

Conventional protein corona theory [19,20] characterises the corona as a state acquired in a single biological fluid, typically serum or plasma, and uses it to interpret opsonisation, clearance, or receptor engagement at one interface. Single-barrier receptor-mediated transcytosis (RMT) models, developed for engineered nanomedicines, describe vesicular transport across one selective interface—most often the BBB—using designed ligands such as anti-TfR antibodies or angiopep-2 conjugates [25,43,44]. Neither framework was developed for environmental particle populations that sequentially encounter chemically distinct biological fluids.

CDBS extends these established concepts in three respects: (i) the corona is treated as a sequential and tissue-stratified property rather than a single equilibrated state, (ii) corona composition is paired explicitly to barrier-specific receptor repertoires, and (iii) translocation across each barrier is framed as a selection problem dependent on the corona–receptor match, rather than as a passive size cut-off. The framework therefore generates testable predictions, the most direct of which is that identical NPs preincubated in matched human chyme, plasma, and intervillous-space fluid should acquire compositionally distinct coronae, with corresponding differences in permeability across paired intestinal, BBB, and placental microphysiological systems, and with selective sensitivity to receptor-targeted blockade (anti-TfR, anti-LRP1, anti-FcRn). None of these predictions has yet been tested experimentally; CDBS should therefore be regarded as a hypothesis-stage organising framework rather than an established mechanism. Table 4 summarises the key differences between conventional protein corona theory, single-barrier receptor-mediated transcytosis models, and CDBS.

Key mechanistic insight (Section 1): Polymer signatures of nanoplastics have been detected in human tissues, but barrier-crossing remains poorly resolved at the mechanistic level; the corona–receptor interface is one underexplored axis along which differential translocation may occur.

Knowledge gap (Section 1): No published study has performed a head-to-head, mechanistically standardised comparison of NP translocation across matched intestinal, BBB, and placental human-derived models using identical particles, identical analytical end-points, and physiologically realistic doses, with appropriately documented contamination controls.

### 1.4. Search Strategy and Structure of the Review

This work is a narrative review with a PRISMA-inspired literature-selection workflow rather than a formal systematic review. Searches were conducted in PubMed, Scopus, and Web of Science, with emphasis on studies published between January 2018 and October 2025. To provide historical and mechanistic context, seminal studies published before 2018 were additionally identified through backward citation tracking, hand-searching of key reviews, the reference lists of foundational articles, and the authors’ own prior work. These earlier publications were retained only when they established key concepts, mechanisms, or methodologies that remain central to understanding nanoplastic interactions with biological barriers. Four search blocks were combined using Boolean operators: (i) exposure block—“nanoplastic*”, “microplastic*”, “MNP*”, “sub-micron polymer particles”; (ii) barrier block—“blood–brain barrier”, “placenta*”, “syncytiotrophoblast”, “intestinal epithelium”, “Caco-2”, “gut barrier”; (iii) mechanism block—“translocation”, “transcytosis”, “endocytosis”, “tight junction”, “protein corona”, “receptor-mediated”; and (iv) tissue-detection block—“human tissue”, “blood”, “brain”, “atherosclerotic plaque”, “biomonitoring”.

Inclusion criteria were: (a) peer-reviewed publication in English with a verifiable DOI; (b) primary experimental data, or explicitly mechanistic conceptual work, relevant to at least one of the three barriers; and (c) sufficient reporting of particle characterisation and dose to allow evidence-level classification. Exclusion criteria were: (a) purely environmental-fate studies without a biological interface; (b) conference abstracts, non-peer-reviewed preprints and predatory-journal outputs; (c) publications lacking a DOI or that could not be independently verified across the three databases; and (d) studies focused exclusively on macroplastic waste or on nanomedicine drug-delivery platforms without an environmental-particle counterpart.

The initial pooled search returned approximately 1650 records. After duplicate removal (~470) and title/abstract screening for topical relevance (~830 excluded), 350 full-text articles were assessed for eligibility. Of these, the primary studies and methodological or conceptual reviews retained for the final synthesis are those cited in the reference list; additional records were consulted as background but not cited to limit redundancy. Each retained mechanistic claim was tagged with an evidence-level label (in silico/in vitro monoculture/in vitro co-culture/animal in vivo/ex vivo human/human in vivo), and the detection–association–mechanism–causality schema was applied at the sentence level. This dual tagging is used throughout Section 3, Section 4, Section 5, Section 6 and Section 7 and consolidated in Table 3.

The structure of the review is as follows. Section 2 covers the physicochemical determinants of NP–membrane interaction and the acquired biological identity conferred by the corona. Section 3, Section 4, and Section 5 present the intestinal, blood–brain, and placental barriers in turn, each with its molecular architecture, candidate transport routes, and the current evidence stratified by model system. Section 6 draws the three barriers together within the CDBS framework. Section 7 reviews shared cellular toxicity mechanisms (oxidative stress, mitochondrial dysfunction, inflammasome activation, genotoxicity, epigenetic remodelling, autophagy failure, and protein aggregation). Section 8 concentrates on the methodological limitations and sources of bias that condition the interpretation of the whole literature. Section 9 outlines a prioritised research agenda; Section 10 states the conclusions. A compact reading path for the review is therefore: Physicochemistry and Corona (Section 2) → Intestinal barrier (Section 3) → Blood–brain barrier (Section 4) → Placental barrier (Section 5) → CDBS integration (Section 6) → Shared toxicity mechanisms (Section 7) → Methodological limits (Section 8) → Research agenda (Section 9) → Conclusion (Section 10).

## 2. Physicochemical Properties and Biomolecular Interactions

### 2.1. Size, Shape, and Surface Charge as Master Variables

Particle diameter, shape, and surface charge govern both diffusion to the membrane and the energetic cost of membrane interaction. Coarse-grained molecular-dynamics (MD) simulations indicate that polystyrene (PS) chains with diameters smaller than the bilayer thickness (~4–5 nm) can partition into the hydrophobic core of model phosphatidylcholine bilayers without producing visible mechanical disruption [45,46]. Above this threshold, penetration is energetically gated by the elastic deformation of the membrane and the desolvation of the particle surface [47,48]. MD studies also suggest polymer-specific permeability rankings that depend on chemistry as well as size—a result that is in silico and not yet validated experimentally in mammalian membrane systems. Larger NPs (>50 nm) are more dependent on shape-mediated effects: rod-like and irregular weathered fragments engage membranes asymmetrically and induce greater bending stress than spheres [46], but virtually all in vitro literature uses commercial PS spheres, creating a systematic bias in the empirical record.

Surface charge is the third master variable. In in vitro experiments, cationic NPs adsorb to anionic plasma-membrane lipids and to the glycocalyx, and produce membrane permeabilisation, mitochondrial depolarisation, and cell death at concentrations at which neutral or carboxylated analogues are tolerated [49,50]. Even nominally “plain” PS NPs acquire a strongly negative zeta potential in aqueous media, owing to surfactant residues and oxidative weathering, and this acquired charge dictates corona composition [21].

### 2.2. Surface Chemistry, Weathering, and the Reality Gap

A central methodological problem of the field is that almost all published in vivo and in vitro NP toxicology relies on pristine, monodisperse, fluorophore-labelled, surfactant-stabilised PS spheres. Environmentally weathered NPs differ in three biologically critical ways: (i) they bear oxidised carbonyl, hydroxyl, and carboxyl groups that increase hydrophilicity and corona affinity [46]; (ii) they leach plasticisers, monomers, and adsorbed pollutants—phthalates, bisphenols, PFAS, polycyclic aromatic hydrocarbons, heavy metals—whose release may dominate the toxicological signature attributed to the polymer [48,51]; and (iii) they are heterogeneous in size and shape. This reality gap is not a peripheral footnote; it constrains the external validity of the entire experimental literature reviewed below and motivates explicit hedging of all claims that originate from pristine-bead studies.

Recent attempts to bridge the reality gap include UV-, ozone-, and mechanical-shear-weathered PS, PE, and PET particle preparations that more closely mimic environmental fragments [48]. Such artificially weathered analogues exhibit altered surface oxygen functional groups, altered protein corona compositions, and altered biological responses compared with pristine PS controls—but standardised, internationally recognised weathered reference materials are still absent [52]. Additional environmental-relevance factors that remain largely unaddressed in toxicology include polymer diversity [9,18], shape diversity (rods, fragments, and fibres rather than spheres), and the presence of co-adsorbed environmental contaminants and microbial biofilms (the “plastisphere”). Until weathered reference materials and standardised exposure protocols become available, dose–response data from pristine-PS studies should be interpreted as upper-bound estimates of biological reactivity rather than as quantitative predictors of human-relevant toxicity.

### 2.3. The Protein and Lipid Corona: Acquired Biological Identity

Within milliseconds of contact with biological fluid, NPs acquire a multilayer corona. The hard corona consists of high-affinity, slowly exchanging proteins (apolipoproteins, immunoglobulins, complement factors); the soft corona is a dynamic, rapidly exchanging outer shell [19,20]. For PS NPs, neutron and small-angle scattering studies suggest hard coronae form fractal-like aggregates of ~1.5–3 protein layers, whose morphology has been correlated with membrane damage in in vitro assays [53]. Cedervall et al. [19] showed that protein adsorption onto sulfonated PS evolves with the surrounding protein concentration, predicting that corona composition in vivo will be highly compartment-dependent.

Kopatz et al. [22] reported, using coarse-grained MD coupled with an oral-gavage mouse experiment, that the composition of the biomolecular corona—not the bare polymer—predicted whether 0.293 µm PS particles partitioned into a model dioleoylphosphatidylcholine bilayer and whether they could be detected in murine brain within 2 h after dosing. The brain-detection arm of this study uses limited animal numbers and warrants independent replication, but the underlying in silico finding directly supports the corona-driven view of barrier engagement. Kihara et al. [21] characterised in detail the lipid composition of coronae formed on PS and PVC NPs in human plasma, reporting preferential adsorption of phosphatidylcholines and lysophosphatidylcholines, with corona evolution over 24 h depending on polymer chemistry.

The corona simultaneously enables and obscures a mechanism: by mimicking endogenous ligands, it can engage transferrin receptor (TfR), low-density lipoprotein receptor-related protein 1 (LRP1), insulin receptor, or apolipoprotein-E receptors—the same machinery exploited by nanomedicines for receptor-mediated transcytosis—but it also creates a moving target for in vitro studies in which foetal bovine serum-derived coronae poorly model human exposure [20].

### 2.4. Interactions with Phospholipid Membranes and Membrane Proteins

All-atom and coarse-grained MD studies converge on three insights, all drawn from in silico model bilayers [46,47,48]: (i) bare NP penetration into a zwitterionic bilayer is energetically gated by water displacement at the lipid–nanoplastic interface; (ii) within the hydrophobic core, PS oligomers can disaggregate and perturb lipid lateral diffusion; (iii) cholesterol-rich, low-fluidity bilayers fracture under nanoplastic-induced stress more readily than fluid POPC bilayers. The biological implication—that BBB endothelial cells, with cholesterol-enriched lipid rafts that scaffold receptor-mediated transcytosis, may be particularly vulnerable to NP-driven mechanical disruption—is plausible but remains in silico and untested in cellular systems.

NP–membrane protein interactions are far less well characterised. Anionic PS NPs have been reported to nucleate α-synuclein fibrillation through binding to the non-amyloid component (NAC) region [36], and more broadly, PS surfaces have been shown by sub-diffraction infrared imaging to template β-sheet-rich amyloid-β conformations in neuronal cells and in cell-free assays, consistent with a catalytic-scaffold role for PS in amyloidogenic protein misfolding [37]. These observations come from in vitro biophysical assays only; their relevance to brain proteinopathy remains a hypothesis rather than a demonstrated mechanism. Analogous templated misfolding of amyloid-β and tau is plausible but not yet experimentally established.

Key mechanistic insight (Section 2): The biologically active entity at the cellular interface is not the bulk polymer but the corona-coated, frequently weathered NP, whose tissue-specific corona composition is one likely determinant of barrier-crossing competence alongside particle size.

Knowledge gap (Section 2): No published study has mapped corona composition simultaneously across matched human chyme/mucus, plasma/CSF, and intervillous-space fluids for environmentally weathered NPs.

## 3. Intestinal Barrier: Molecular Transport Mechanisms

**Figure 2:**
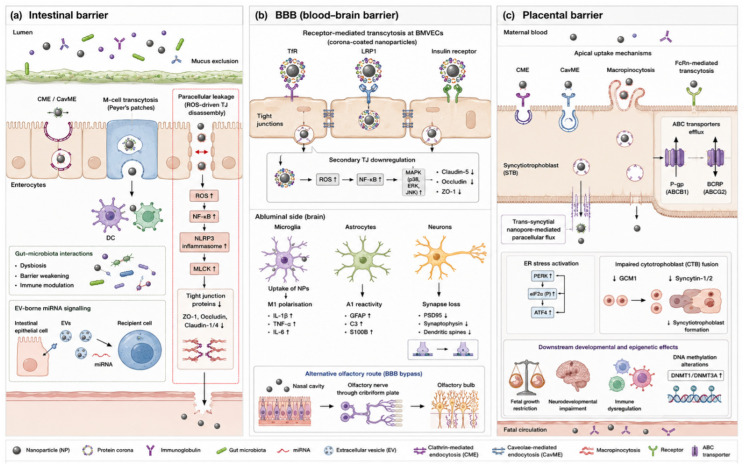
Tissue-specific translocation mechanisms across intestinal, blood–brain, and placental barriers. (**a**) Intestinal barrier. Luminal nanoplastics (NPs) face mucus exclusion before enterocyte uptake by CME/CavME or M-cell transcytosis at Peyer’s patches. Paracellular leakage arises secondarily via a ROS → NF-κB → NLRP3 → MLCK cascade that disassembles tight junctions (ZO-1, occludin, claudin-1/4↓). Parallel gut-microbiota dysbiosis and EV-borne miRNA signalling are shown. (**b**) Blood–brain barrier. Corona-coated NPs engage BMVECs through receptor-mediated transcytosis (TfR, LRP1, insulin receptor); secondary TJ downregulation proceeds through ROS → NF-κB → MAPK signalling. Abluminal NP uptake drives microglial M1 polarisation, A1 astrocyte reactivity and neuronal synapse loss. An alternative olfactory route bypasses the BBB via the cribriform plate. (**c**) Placental barrier. Apical uptake at the syncytiotrophoblast (STB) occurs through CME, CavME, macropinocytosis and FcRn-mediated transcytosis, counterbalanced by P-gp/BCRP efflux and supplemented by trans-syncytial nanopore-mediated paracellular flux. NP-induced PERK/eIF2α/*ATF4* ER stress suppresses *GCM1* and syncytin-1/2, impairing cytotrophoblast (CTB) fusion. Downstream effects include foetal growth restriction, neurodevelopmental impairment, immune dysregulation, and DNMT1/DNMT3A-mediated DNA methylation alterations. Created with BioRender.com. Abbreviations: BMVECs, brain microvascular endothelial cells; TfR, transferrin receptor; LRP1, low-density lipoprotein receptor-related protein 1; ROS, reactive oxygen species; NF-κB, nuclear factor kappa-light-chain-enhancer of activated B cells; MAPK, mitogen-activated protein kinase; NLRP3, NOD-like receptor family pyrin domain containing 3; MLCK, myosin light chain kinase; TJ, tight junction; ZO-1, zonula occludens-1; CME, clathrin-mediated endocytosis; CavME, caveolae-mediated endocytosis; EVs, extracellular vesicles; miRNA, microRNA; STB, syncytiotrophoblast; CTB, cytotrophoblast; FcRn, neonatal Fc receptor; P-gp, P-glycoprotein; BCRP, breast cancer resistance protein; PERK, PKR-like endoplasmic reticulum kinase; eIF2α, eukaryotic initiation factor 2 alpha; *ATF4*, activating transcription factor 4; *GCM1*, glial cells missing homolog 1; DNMT1/3A, DNA methyltransferase 1/3A. Arrows indicate the direction of nanoparticle transport, translocation, intracellular trafficking, or downstream biological events, depending on the pathway illustrated.

Figure 2a illustrates the intestinal translocation routes discussed in this section; sub-panels (b) and (c) show blood–brain barrier and placental mechanisms addressed in Section 4 and Section 5 respectively. The intestinal epithelium is the principal port of entry for ingested NPs. Estimated daily exposure ranges from ~10^4^ to >10^5^ particles per person from food, beverages, and incidental dust ingestion, with substantial uncertainty driven by methodological variation between exposure-assessment studies [54,55,56]. Barrier function depends on a regulated interplay of mucus exclusion, transcellular endocytosis, paracellular tight junctions, microfold-cell sampling at Peyer’s patches, and the resident microbiota.

### 3.1. Transcellular Uptake: Clathrin- and Caveolin-Mediated Endocytosis

In differentiated Caco-2 enterocyte monolayers, 40–100 nm PS NPs are internalised by a combination of clathrin-mediated endocytosis (CME), caveolin-mediated endocytosis (CavME), and macropinocytosis [57,58]. Selective inhibitor studies—chlorpromazine for CME, genistein and methyl-β-cyclodextrin for CavME, and cytochalasin D for macropinocytosis—indicate that no single pathway accounts for total uptake, consistent with a corona-dependent, multi-receptor entry. CME generates ~70–150 nm coated vesicles that fuse with early endosomes and traffic to lysosomes (Rab7+/LAMP2+), where prolonged retention has been documented for PS NPs in vitro [33]. CavME, by contrast, may bypass lysosomal degradation, providing a route for sustained intracellular accumulation [58].

In a 32-week chronic exposure study in mice, drinking-water PS-NP at 0.1–10 mg L^−1^ produced parallel upregulation of clathrin and caveolin-1 in intestinal tissue [28]. Whether this represents adaptive compensation or pathological vesicle storm is unresolved. A critical caveat applies: the 0.1–10 mg L^−1^ dosing is several orders of magnitude above the most commonly cited estimates of human drinking-water exposure (typically tens of particles per litre); the molecular response signature may therefore not be transferable to human-relevant doses (see Section 8.3).

### 3.2. Paracellular Transport: Tight-Junction Disruption

The most consistently reported intestinal effect of PS NPs and MPs in animal models is downregulation of zonula occludens-1 (ZO-1), occludin (OCLN), and claudin-1 (CLDN-1). Liang et al. [27] reported that PS particles of 50 nm, 500 nm and 5 µm all reduced TJ-protein expression in mouse colon, with the largest particles producing the most severe damage—a counter-intuitive finding most plausibly explained by indirect, oxidative-stress-mediated TJ disruption rather than direct paracellular flux. Mechanistically, the same study implicated the ROS → NF-κB/NLRP3/IL-1β axis and myosin-light-chain kinase (MLCK), with rescue by N-acetylcysteine (NAC), MCC950 (NLRP3 inhibitor) and ML-7 (MLCK inhibitor) in in vitro Caco-2 monolayers. The data support a coherent ROS → NF-κB → NLRP3 → MLCK → TJ-disassembly cascade in this experimental system, but they do not establish the cascade in human intestinal mucosa.

A discordant observation comes from Domenech et al. [26], who exposed differentiated human Caco-2 monolayers to 30 nm PS NPs for 8 weeks and found accumulation but minimal genotoxicity, oxidative DNA damage, or stress-gene dysregulation. Possible reasons for the discrepancy with murine data include (i) inter-species TJ regulation differences, (ii) the absence of mucus, microbiota and immune cells in monocellular models, and (iii) the 1–3-orders-of-magnitude dose differential between rodent and in vitro studies. A 2025 follow-up study reported that PS NPs do not directly cause single- or double-strand breaks (γH2AX and comet assays both negative) but downregulate base-excision-repair and double-strand-break-repair genes, suggesting sublethal genome instability rather than overt genotoxicity [29]. In primary human peripheral-blood lymphocytes, Berber et al. [59] reported reduced mitotic index and dose-dependent increases in micronucleus frequency and comet tail length over 0.001–100 µg/mL, consistent with overt cytogenotoxicity in a proliferating cell system (further discussed in Section 7.4). The non-mutagenic but DNA-repair-suppressing pattern of the Caco-2 work has regulatory implications because it would fail standard genotoxicity assays yet may amplify the impact of co-exposure to genotoxic pollutants—an important nuance that earlier reviews have not consistently captured.

### 3.3. M-Cell Uptake and Antigen Sampling

Microfold (M) cells overlying Peyer’s patches sample particulates from the lumen and deliver them to underlying dendritic cells. in vitro co-cultures of Caco-2 with Raji B cells (which induce M-cell-like phenotypes) consistently show enhanced uptake of 50–500 nm particles compared with monoculture [60,61]. Direct evidence of M-cell-mediated NP transcytosis in humans is lacking; this route is mechanistically plausible and is a candidate portal for systemic NP dissemination but should not be treated as established.

### 3.4. NP–Microbiota Interactions

The gut microbiota functions as a fourth layer of intestinal defence. Multiple murine studies report compositional shifts after PS-MP or PS-NP exposure—typically a decrease in Bacteroidetes and *Akkermansia muciniphila*, an increase in Firmicutes and Proteobacteria, and reductions in short-chain-fatty-acid producers [62,63,64]. Hsu et al. [30] reported in mice and Caco-2 co-culture that PS NPs alter intestinal extracellular vesicle microRNA cargo, providing a candidate, though not yet replicated, route by which luminal NP exposure reprograms host–microbe communication.

In a cross-sectional human study of 50 inflammatory bowel disease (IBD) patients and 52 healthy controls, Yan et al. [31] reported that faecal MP burden was ~50% higher in IBD patients (41.8 vs. 28.0 items g^−1^ dry weight) and tracked disease-activity scores. The direction of causality—NPs promoting IBD versus IBD-associated retention of NPs—cannot be inferred from this design and remains an open question.

### 3.5. Signalling Pathway Alterations

NP-exposed enterocytes, in animal and in vitro studies, activate NF-κB-driven proinflammatory transcription (TNF-α, IL-6, IL-8); NLRP3-inflammasome assembly with IL-1β secretion; MAPK signalling (ERK1/2, p38); and PERK/*ATF4*/CHOP-mediated endoplasmic reticulum (ER) stress with caspase-3-dependent apoptosis [27,65]. The PERK arm is conserved with that observed in placental trophoblasts (Section 5.3), suggesting a tissue-conserved ROS → ER stress → integrated stress response axis. Conservation of pathway, however, is not the same as conservation of consequence; tissue-specific repair capacities differ markedly (see Section 6.4).

Key mechanistic insight (Section 3): Intestinal barrier compromise is best conceptualised—within the available animal evidence—as an ROS-initiated, NF-κB/NLRP3/MLCK-amplified loop that secondarily disrupts TJ integrity, rather than a direct mechanical breach by NPs. In human Caco-2 exposures, the dominant signature at lower and chronic doses appears to be sublethal genome instability rather than overt cytotoxicity.

Knowledge gap (Section 3): Human-relevant doses, integrated mucus–microbiota–epithelial–immune in vitro systems, and longitudinal studies linking dietary NP intake to TJ and microbiome biomarkers in humans are largely absent; the IBD–MP correlation remains causally ambiguous. Throughout this section, findings from Caco-2 monocultures should be read as isolated-epithelium mechanistic evidence and are not equivalent to observations in mucus- and microbiota-inclusive intestine-on-chip platforms or in rodent gavage studies; Table 5 disaggregates the key intestinal studies by model system, dose, and dose-realism to support this distinction.

## 4. Blood–Brain Barrier: Mechanistic Hypotheses and Evidence Levels

In contrast to the intestinal epithelium, which presents a chyme-facing apical surface with high luminal exposure but rapid epithelial turnover, the BBB is a tightly sealed endothelial interface with limited regenerative capacity and a distinct junctional and transcytotic repertoire. The BBB is composed of brain microvascular endothelial cells (BMVECs) sealed by a claudin-5-dominated tight junction with claudin-3, claudin-12, occludin, ZO-1/ZO-2, and JAM-A, supported by pericytes, astrocyte end-feet, and a continuous basement membrane [23]. BBB disruption is recognised as an early event in neurodegeneration, but the strength of evidence for nanoplastic-driven BBB disruption varies sharply between in silico, animal, and human studies, and warrants explicit stratification.

### 4.1. In Silico and Animal Evidence: Particles Can Reach Brain Tissue

In an animal in vivo experiment, Shan et al. [32] reported that 50 nm PS NPs administered orally to mice (0.5–50 mg kg^−1^ for 7 days) accumulated dose-dependently in brain parenchyma, increased BBB permeability (Evans blue assay), and were detected within microglia. Kopatz et al. [22], again in mice and supported by coarse-grained MD, reported that 0.293 µm PS particles reached the brain within 2 h of a single oral gavage, while 1.14 µm and 9.55 µm particles did not, attributing the size selectivity to corona-mediated lipid-bilayer partitioning. Both studies use small animal numbers and aggressive dosing, and neither has been independently replicated in mice from a different vendor or with a different commercial particle batch. The size cut-off and the corona–lipid bilayer mechanism are consequently best treated as promising but provisional.

Earlier, Deng et al. [66] reported tissue accumulation of PS MPs in mouse liver, kidney, and gut. This study has been the subject of substantive published critique on the plausibility of the high reported tissue concentrations and the absence of contamination controls [67,68], and we cite it here primarily to illustrate the pattern of contested early evidence rather than as an established mechanistic finding.

### 4.2. Human Evidence: Detection, Association, Mechanism, and Causality

For clarity throughout this section we apply four distinct evidence categories: detection (a polymer signature has been chemically identified in the tissue, subject to analytical limitations); association (statistical correlation between exposure or burden and a clinical or biological endpoint, with no temporal or mechanistic resolution); mechanism (an experimentally demonstrated molecular pathway that could connect exposure to outcome, typically from cellular or animal data); and causality (a tested causal relationship that excludes confounding and reverse causation, typically requiring intervention or longitudinal study with sufficient power). Most current human nanoplastic evidence is detection-class; some studies report association; the mechanism is largely extrapolated from non-human systems; and causality has not been established for any human disease endpoint.

The most discussed human evidence is the brain-tissue dataset of Nihart et al. [18], who applied Py-GC/MS, attenuated-total-reflectance Fourier-transform infrared spectroscopy, and energy-dispersive electron microscopy to 52 frontal cortex autopsy samples. They reported median MNP mass concentrations of approximately 4900 µg g^−1^ in 2024 brains versus ~3300 µg g^−1^ in 2016 brains, identified PE as the dominant polymer, and observed nanoscale shard-like fragments by EM. Brains from individuals who died with documented dementia diagnoses carried 3- to 5-fold higher MNP signals than non-dementia controls. Classified within the framework above, this is detection-class evidence for the polymer signature (with caveats below) and association-class evidence for the dementia link; it is not mechanistic or causal evidence.

Several caveats apply: (i) Py-GC/MS quantifies polymer-specific pyrolysis fragments and is sensitive to lipid co-pyrolysis interference, particularly in lipid-rich tissues; the magnitude of mass concentrations reported has been independently questioned, with arguments that lipid-derived alkadienes and styrene-mimetic peptide pyrolysates may inflate apparent PE and PS signals in lipid-rich tissue [3,4]. (ii) The dementia association is cross-sectional and cannot establish whether elevated MNP load drives neurodegeneration, accumulates because of impaired clearance in dementia, or is confounded by shared lifestyle or vascular factors; reverse causation (impaired BBB function and lymphatic clearance in dementia leading to particle retention) is at least as plausible as the forward direction. (iii) Procedural blanks are essential to interpret tissue concentrations of this magnitude [5,6] and the contamination risk during lipid-rich brain processing is particularly high. (iv) The number of decedent brain donors is modest and the comparison between the 2016 and 2024 cohorts could be confounded by storage time or processing differences. Garcia et al. [9], using the same analytical platform on placental tissue, reported tissue burdens of 6.5–790 µg g^−1^, illustrating the spread of tissue-specific apparent loads. The brain finding is important and warrants urgent independent replication, but should not be cited as established quantitative human exposure data without explicit acknowledgment of these limitations.

A second prominent human dataset is the cardiovascular study of Marfella et al. [14], in which 304 patients undergoing carotid endarterectomy were prospectively followed for 34 months; detection of MNPs in excised plaque was associated with a hazard ratio of 4.53 (95% CI 2.00–10.27) for myocardial infarction, stroke, or all-cause death. This is association-class evidence, not causal: although the prospective design rules out reverse causation for the event, MNP burden could be a marker of upstream behavioural, metabolic, or environmental factors that themselves drive plaque vulnerability, and the single-centre design has not been independently replicated. The authors acknowledge the contamination-control limitations inherent to plaque processing. Marfella et al. [14] is the first study to associate MNP detection with a hard clinical endpoint, and merits major follow-up, but cannot be cited as evidence that nanoplastics cause cardiovascular events.

### 4.3. Candidate Transport Mechanisms

Four routes are mechanistically plausible at the BBB. We outline them below with explicit distinctions between the molecular biology established in nanomedicine and drug-delivery literature—where these receptors have been extensively characterised—and the more limited evidence specifically implicating them in nanoplastic translocation.

(i) Receptor-mediated transcytosis (RMT) via the transferrin receptor (TfR1). TfR1 is abundantly expressed on the luminal membrane of brain microvascular endothelial cells (BMVECs) and is one of the most extensively characterised BBB transcytosis receptors [25,43,69,70]. Nanomedicine studies have shown that transferrin- or anti-TfR-antibody-decorated nanoparticles can cross the BBB by RMT, with parenchymal delivery efficiency dependent on receptor avidity: high-affinity bivalent ligands are sequestered in lysosomes, whereas low-affinity or pH-sensitive linkages favour transcytosis [43,71,72,73]. Whether plasma-corona-coated NPs that adventitiously display transferrin engage TfR1 with comparable efficiency in vivo has not been directly demonstrated, and TfR1-mediated NP transcytosis at the BBB remains hypothetical.

(ii) Low-density lipoprotein receptor-related protein 1 (LRP1)-mediated transcytosis. LRP1 is constitutively expressed in BMVECs and is the canonical efflux receptor for amyloid-β clearance from the brain [74,75]. LRP1 binds ApoE-, ApoJ-, α2-macroglobulin-, and tPA-decorated ligands—molecules likely to be enriched in plasma-derived NP coronae—and has been engineered as an entry receptor for angiopep-2-conjugated nanocarriers that cross the BBB without requiring strong cargo acidification [25,44,76]. Direct experimental evidence that nanoplastic coronae engage LRP1 to cross the BBB is currently lacking.

(iii) Caveolin-mediated transcytosis. Caveolae are 60–80 nm cholesterol- and caveolin-1-rich flask-shaped invaginations abundant in BMVECs [25,76]. Caveolar transcytosis is a non-clathrin, dynamin-dependent route that can bypass lysosomal degradation; caveolar biogenesis and stability are regulated by cavin-1/PTRF and by Src-family-kinase-mediated phosphorylation of caveolin-1 at Tyr14 [77,78]. Kuhn et al. [58] showed that the relative contribution of CME and CavME to nanoparticle uptake is cell-type-specific and depends on particle physicochemistry; for PS NPs, both pathways operate in epithelial and endothelial cells.

(iv) Adsorptive transcytosis. Cationic NPs or cationised coronae interact with the negatively charged glycocalyx and trigger non-specific vesiculation; this route is concentration-dependent and saturates at high doses, with limited specificity [25].

(v) Paracellular leakage secondary to TJ disruption. Shan et al. [32] reported in hCMEC/D3 endothelial monolayers that 50 nm PS NPs induced ROS, NF-κB activation, TNF-α secretion, and necroptosis—mechanisms consistent with secondary TJ opening rather than direct paracellular flux of the particle itself. This evidence is in vitro and would benefit from replication in iPSC-derived BBB-on-chip systems with physiological shear stress [79,80].

(vi) Olfactory bulb route bypassing the BBB. Amato-Lourenço et al. [15] detected MNPs in 8 of 15 human olfactory bulbs by µ-Raman, identifying PE, PP, and nylon fibres at sizes down to ~5.5 µm. The olfactory route—direct nose-to-brain transport via the cribriform plate—has been demonstrated for ultrafine particles in rodent models and is mechanistically plausible for inhaled NPs in humans, though direct quantitative evidence of NP transit across the human cribriform plate remains absent. Given that inhalation is a substantial component of total NP exposure [54,56], this route may be quantitatively more important than transbarrier blood–brain transport.

### 4.4. Tight-Junction Alterations: Conserved Mechanism, Variable Strength of Evidence

In hCMEC/D3 monolayers, PS NPs reduce claudin-5 and ZO-1 expression in an ROS-dependent fashion, with NAC pretreatment abolishing barrier hyperpermeability [32]. The MAPK/ERK arm has been shown to regulate claudin expression and distribution in mouse barrier epithelia [81]. These mechanisms parallel the intestinal-barrier cascade described in Section 3.2. They are in vitro and animal findings; the strength of evidence is moderate.

At the molecular level, claudin-5 turnover at the BBB is regulated by phosphorylation-driven endocytosis and trafficking. PKC- and Rho-kinase-dependent phosphorylation of serine/threonine residues promotes claudin-5 dissociation from ZO-1 and its endocytic internalisation via clathrin- and lipid-raft-dependent routes, with subsequent sorting between recycling and lysosomal degradation determining whether the junction is transiently opened or persistently disrupted [82,83]. ZO-1 trafficking is regulated by the same kinase network and by tyrosine phosphorylation that disrupts its scaffolding interactions with occludin and the actin cytoskeleton, allowing junctional unzipping [84,85]. NP-driven ROS oxidise redox-active cysteines in junctional proteins and activate PKC and Src-family kinases, providing a plausible coupling between the upstream ROS signal and downstream junctional internalisation; direct biochemical demonstration of this coupling for nanoplastics is still lacking.

### 4.5. Microglial Activation and Neuroinflammatory Hypotheses

Animal evidence implicates microglia as the principal cellular amplifier of NP neurotoxicity. PS NPs are preferentially phagocytosed by microglia over astrocytes or neurons in mixed cultures and in mouse brain [33,86]. PS-NP-laden microglia have been reported to adopt an M1 (pro-inflammatory) phenotype with concomitant astrocyte A1 reactivity, synapse loss, and hippocampal-neuron damage that can persist for at least 10 months after a 1-week exposure [34]. Inhibition of microglial activation by minocycline rescued synapse density, providing a mechanistic link in this model system between nanoplastic-driven neuroinflammation and structural neurodegeneration. We emphasise that this is animal evidence at high doses; its translation to human chronic, low-level NP exposure is a hypothesis requiring direct testing.

In Alzheimer’s disease (AD) models, anionic PS NPs aggravated disease progression in APP/PS1 mice with accompanying peripheral immunometabolic perturbations [35]. Anionic PS NPs nucleate α-synuclein and amyloid-β aggregation in vitro by adsorbing onto specific structural motifs [36,37]. The inference that NP surfaces could accelerate human proteinopathy is mechanistically attractive but currently rests on biophysical in vitro assays only and should be presented as a testable hypothesis rather than a demonstrated pathway.

### 4.6. Mitochondrial Dysfunction

In BMVECs and other cell types, PS NPs reduce mitochondrial membrane potential, deplete ATP, increase mitochondrial ROS, and upregulate Bax/Bcl-2 ratios consistent with intrinsic apoptosis [87]. The hierarchical oxidative-stress paradigm—Tier 1 Nrf2/HO-1 antioxidant defence, Tier 2 redox-sensitive MAPK/NF-κB inflammation, Tier 3 mitochondrial dysfunction and apoptosis—maps cleanly onto BMVEC and other tissue responses [88].

Key mechanistic insight (Section 4): Animal and in vitro data are consistent with BBB breach by NPs through corona-dependent RMT in the small-particle regime (<300 nm) and through secondary paracellular leakage following ROS-driven TJ disassembly at higher doses. Microglia, not endothelial cells, appear to be the principal site of intracerebral persistence in animal models. The olfactory route deserves greater attention than it has received and may dominate inhaled NP exposure.

Knowledge gap (Section 4): No published study quantitatively links circulating NP load to BBB-permeability biomarkers (S100B, neurofilament-light-chain) in humans. The relative contributions of olfactory, hematogenous, and lymphatic routes to the brain MNP burden reported by Nihart et al. [18] remain unallocated, and the dementia association requires independent cohort replication with rigorous contamination controls.

### 4.7. Model-System Contrast: iPSC-Derived BBB Chips Versus In Vivo Animal Work

Mechanistic insights on nanoplastic–BBB interactions now arise from two very different platforms and the two should not be conflated. Traditional in vivo rodent studies (gavage, inhalation, intravenous) report region-specific brain accumulation, glial activation, and behavioural change, but they aggregate all barrier-crossing routes (BBB, blood-CSF, olfactory) and rely on fluorescent tracer detection, which is subject to leached-dye artefacts and to labelling-dependent surface chemistry. Human iPSC-derived brain microvascular endothelial cell (BMVEC) chips and brain-chip platforms, in contrast, isolate the BBB compartment, express the endothelial claudin-5 tight-junction phenotype, and permit label-free readouts of TEER and paracellular tracer permeability. They do not, however, reproduce systemic pharmacokinetics, and their mechanistic conclusions apply to the endothelial layer alone rather than to whole-brain outcomes. We therefore treat findings from iPSC-BBB chips as high-resolution mechanistic evidence for the endothelial component, and rodent in vivo findings as lower-resolution but organism-level evidence; the two are complementary rather than substitutable.

A number of contradictory or contested findings warrant explicit acknowledgement. The magnitude of PS bead penetration into intact rodent brain reported in early in vivo studies has been questioned on the grounds that free fluorophore leached from the particle can accumulate in lipid-rich brain regions independently of the polymer, so that fluorescence in tissue does not equate to polymer in tissue. The absolute mass concentrations reported by Nihart et al. [18] have been argued to be inflated by lipid co-pyrolysis on Py-GC/MS [3,4]. Reported BBB transwell permeability of PS NPs varies by more than an order of magnitude between laboratories using nominally similar cell lines and doses, in part because of surface chemistry and corona formation differences that are rarely reported in enough detail to reproduce. These contradictions do not negate the field’s conclusion that NP-BBB interaction is a legitimate mechanistic concern; they do argue for orthogonal confirmation before any specific quantitative claim is accepted.

## 5. Placental Barrier: Molecular Transfer

The placental syncytiotrophoblast differs again from the intestinal and BBB interfaces: it lacks lateral junctions and yet displays size-dependent paracellular permeability, and it operates bidirectionally to regulate maternal–foetal exchange across a relatively short gestational window. The human placenta interposes a single-cell-thick syncytiotrophoblast—formed by fusion of cytotrophoblast cells, lacking lateral junctions, polarised between an apical brush-border facing the maternal intervillous space and a basal membrane facing the foetal capillary endothelium—between maternal and foetal circulations. This unusual architecture poses both an obstacle and a permissive route for NP transfer.

### 5.1. Ex Vivo and In Vitro Translocation: Size-Dependent and Energy-Dependent

The dual ex vivo perfused human placental cotyledon model is the most physiologically realistic system available. Wick et al. [38] first reported size-dependent maternal-to-foetal translocation of fluorescent PS particles, with 50, 80, and 240 nm beads crossing, but 500 nm beads excluded. Grafmueller et al. [39] extended this to bidirectional transfer of plain and carboxylated 50–300 nm PS particles, finding that foetal-to-maternal flux exceeded maternal-to-foetal flux—a result inconsistent with passive diffusion (the reference paracellular tracer antipyrine showed the expected delayed F→M flux) and instead pointing to active, energy-dependent transcellular transport. All particles accumulated in the syncytiotrophoblast irrespective of perfusion direction, identifying this layer as the principal regulator of NP transfer. These studies are central to the field but depend on fluorescent labelling, which carries known artefactual concerns [89,90]; a label-free re-examination of the size cut-off would substantially strengthen the field. Table 6 summarises the bidirectional transfer findings across placental model systems, and highlights the weathered/additive-loaded fragment data gap.

In the BeWo b30 trophoblast monoculture, 50 nm PS particles were reported to cross the trophoblast barrier ~6-fold more efficiently than 100 nm analogues [91]. Aengenheister et al. [40], in a BeWo–HPEC-A2 co-culture, found limited translocation of 49 nm PS NPs and no translocation of 70 nm PS NPs, illustrating the considerable inter-laboratory variability that pervades the field. Different cell-line clones, membrane pore sizes, and serum sources produce divergent quantitative answers from nominally similar models.

More recent work using ACH-3P, BeWo b30, Jeg-3, and JAR cell lines reports translocation of PS NPs and decreased trophoblast viability, motility, and tubule formation, with elevated ROS in human umbilical-vein endothelial cells [41]. Ragusa et al. [92] reported, in cultured human trophoblasts, that PS NPs induced lysosomal destabilisation, autophagic blockade, and ER- and mitochondria-coupled cytoplasmic alterations—an in vitro recapitulation of the ER stress and mitochondrial pathology that Ragusa et al. [93] observed in ex vivo human placentas.

### 5.2. Transport Pathways and Trophoblast Receptors

The syncytiotrophoblast has no classical paracellular junctions, yet exhibits size-dependent paracellular permeability. This paradox is at least partially resolved by trans-syncytial nanopores (TSNs)—membrane-bound channels visualised by serial-block-face SEM at densities of ~10^7^ cm^−3^ of tissue, sufficient to account for measured paracellular fluxes [24]. Whether TSNs admit corona-coated NPs is mechanistically plausible but unproven.

Transcellular routes implicated for NPs draw on the same fundamental endocytic and transcytotic machinery characterised in placental drug-transport biology:

Clathrin- and caveolin-mediated endocytosis at the apical brush-border microvilli internalises cargo via 70–150 nm clathrin-coated vesicles and 60–80 nm caveolae, respectively; these routes are responsible for the bulk uptake of plasma proteins, lipoproteins, and a range of nanoscale therapeutics in in vitro trophoblast and primary cytotrophoblast studies [58,91]. Macropinocytosis has been demonstrated for PS NPs in primary trophoblasts.

FcRn-mediated transcytosis is the molecularly best-characterised transcellular route in the human placenta. The neonatal Fc receptor (FcRn) is a non-classical MHC-class-I-like heterodimer of an α-chain and β2-microglobulin, expressed in intracellular endosomes of the syncytiotrophoblast, where it binds IgG at acidic endosomal pH (~6) and releases it at neutral basolateral pH, mediating the unidirectional maternal-to-foetal transfer of IgG that is the basis of neonatal passive immunity [94,95,96,97,98]. Whether nanoplastic coronae acquire IgG of sufficient density and orientation to hijack this pathway has not been tested experimentally; FcRn-mediated NP transfer therefore remains a candidate mechanism rather than a documented one.

ABC-transporter-mediated efflux counterbalances apical-to-basal transfer at the placenta. P-glycoprotein (P-gp, ABCB1) and breast cancer resistance protein (BCRP, ABCG2) are highly expressed at the apical (maternal-facing) membrane of the syncytiotrophoblast throughout gestation; their function is to expel xenobiotics, drugs, and metabolites back into the maternal circulation, protecting the foetus [99,100,101,102,103]. BCRP/ABCG2, in particular, is most abundantly expressed in the human placenta among all tissues [104]. The asymmetric foetal-to-maternal > maternal-to-foetal flux of PS NPs reported by Grafmueller et al. [39] is consistent with active ABC-transporter-mediated efflux, but no published study has directly demonstrated that nanoplastics are substrates for P-gp or BCRP, only that the flux asymmetry is observed. Reduced expression of placental P-gp and BCRP in conditions such as preeclampsia, gestational diabetes, and intrauterine infection [105,106] could in principle increase foetal NP exposure in vulnerable pregnancies, but this remains a hypothetical risk pathway.

### 5.3. Syncytialisation Failure and the PERK/eIF2α/ATF4 Axis

Cheng et al. [42] reported that gestational PS-NP exposure activates the PERK/eIF2α/*ATF4* arm of the unfolded-protein response in mouse placenta and human trophoblasts, suppressing *GCM1* and syncytin expression and impairing cytotrophoblast → syncytiotrophoblast fusion, with downstream increases in embryo resorption. The molecular logic fits the well-characterised biology of the integrated stress response, in which PERK phosphorylates eIF2α to attenuate cap-dependent translation while selectively permitting translation of *ATF4*, which in turn transactivates CHOP, GADD34, ATF3, and the amino-acid-response gene network [107,108]. In the placenta specifically, translation attenuation is particularly costly because syncytialisation requires high-throughput synthesis of *GCM1*, syncytin-1, and syncytin-2. Cheng et al. [42] provide a candidate link between NP-induced ER stress and pregnancy failure that is broadly consistent with the conserved ROS → ER-stress axis seen at other barriers, and that mirrors the ER ultrastructural pathology in Ragusa et al. [93]. Independent replication in an additional rodent model and a human placenta-on-chip system would substantially strengthen this mechanism.

### 5.4. Detection in Human Placenta: Strengths and Limits

Ragusa et al. [8] used µ-Raman to detect ~10 µm PP-dominated MP fragments in four of six human placentas—a landmark observation tempered by the small sample size and the practical Raman lower limit of ~1 µm. Garcia et al. [9], using Py-GC/MS in 62 placentas, reported polymer signatures in all samples with concentrations of 6.5–790 µg g^−1^; PE accounted for ~54% of total polymer mass, with PVC and nylon each ~10%. Py-GC/MS quantifies polymer mass and is robust to optical detection limits but is destructive and offers no size or count information; the same lipid-co-pyrolysis interferences that affect brain measurements (Section 4.2) apply to placenta. The detection of polymer signatures in placenta is now well replicated; quantitative tissue concentrations should be interpreted with explicit acknowledgment of analytical limits.

### 5.5. Developmental and Epigenetic Consequences

Maternal NP exposure during gestation has been associated, in mouse and rat models, with reduced foetal weight, hepatic and testicular toxicity in male offspring, neurodevelopmental impairment, and altered DNA methylation patterns [109,110]. PE-MP exposure has been reported to increase global DNA methylation in adult and offspring testicular tissue in rodent models [111], and MPs can act as vectors for endocrine-disrupting compounds (e.g., bisphenol A) that upregulate DNMT1 and DNMT3A in vitro [112]. Intratracheal exposure of late-pregnant rats to 20 nm PS NPs led to foetal deposition in the placenta, foetal liver, lung, heart, kidneys, and brain [113], establishing a maternal-respiratory-to-foetal route in addition to the oral one. All these findings are from animal in vivo; their translation to human pregnancy outcomes is plausible but unproven.

Key mechanistic insight (Section 5): The syncytiotrophoblast is not a passive sieve but an active, ER-stress-vulnerable interface where NPs converge on the PERK/eIF2α/*ATF4* axis to disrupt syncytialisation, with consequences extending beyond passive transfer to placental function itself—a finding whose human relevance now constitutes a high-priority research target.

Knowledge gap (Section 5): No human study has correlated maternal blood NP burden with placental NP load, foetal cord-blood NP concentrations, pregnancy outcomes, and offspring neurodevelopment in the same cohort—an integrative gap that limits clinical inference.

### 5.6. Foetal Exposure: What Is and Is Not Known

Because placental outcomes are of particular public concern, the current state of knowledge is worth stating explicitly. What is known: Polymer signatures have been reported in term human placenta by vibrational spectroscopy [8] and by Py-GC/MS [9]; ex vivo perfusion and in vitro BeWo and primary trophoblast systems demonstrate that PS particles below ~200 nm can cross to the foetal side under experimental conditions [8,9]; and PS-induced PERK/eIF2a/*ATF4* signalling in trophoblasts is reproducible across independent laboratories (Section 5.3). What is not known: Whether meaningful numbers of environmentally weathered NPs cross the human placenta at real-world exposure levels; whether any foetal-tissue accumulation persists beyond gestation; whether the syncytialisation phenotypes observed in vitro translate to human pregnancy outcomes; and whether specific polymer types, sizes, or additive loads differ in transfer efficiency in humans. Current evidence is therefore limited, primarily experimental, and does not yet support quantitative statements about foetal exposure or foetal risk in the general population.

## 6. Comparative Molecular Vulnerability and the CDBS Framework

### 6.1. Junctional Architecture

The intestinal epithelium and the BBB share a tight-junction-based paracellular seal whose molecular composition differs sharply [23,114,115]. The BBB is dominated by claudin-5 with claudin-3 and claudin-12, occludin, ZO-1/ZO-2, and JAM-A, producing trans-endothelial electrical resistances >1500 Ω·cm^2^ in rodent brain in vivo. The small intestine expresses a heterogeneous claudin profile (claudin-1, -3, -4, -7, -15) optimised for selective ion permeability with TEER values in the 200–400 Ω·cm^2^ range. The placental syncytiotrophoblast, lacking lateral junctions altogether, deploys trans-syncytial nanopores [24] and a polarised brush-border for transcellular regulation. NPs therefore encounter qualitatively different gatekeeping at each barrier, and a finding of “tight-junction disruption” in one cannot be naively extrapolated to another.

### 6.2. Transcytotic Machinery Across Barriers

BMVECs express high densities of TfR, LRP1, insulin receptor, basigin, and abundant caveolae, the receptors most readily engaged by ApoE-, transferrin-, or albumin-coated coronae. The intestinal enterocyte preferentially uses CME and macropinocytosis. The syncytiotrophoblast deploys CME, CavME, FcRn-mediated transcytosis (for IgG), and ABC-transporter-mediated efflux (P-gp, BCRP) that asymmetrically polarises particle handling toward the maternal side. This barrier-specific receptor heterogeneity provides the rationale for the CDBS prediction outlined in Section 1.3, namely that compartment-matched NP coronae should engage these receptor repertoires differentially; the prediction remains experimentally untested.

### 6.3. Oxidative-Stress Sensitivity and Repair

All three barriers respond to NP load with ROS generation, NF-κB activation, and pro-inflammatory cytokine release in in vitro and animal studies. The BBB endothelium is particularly susceptible because cerebral metabolism is energy-intensive and antioxidant reserves at the endothelial layer are limited. Placental trophoblasts are similarly ROS-vulnerable, particularly via PERK/eIF2α/*ATF4* ER stress. The intestinal epithelium has higher baseline antioxidant capacity (Nrf2-driven GSH and SOD pools) but is exposed to far higher luminal NP concentrations and microbiota-derived secondary stressors. Repair capacity differs sharply between barriers: enterocytes turn over within 3–5 days, allowing rapid replacement; the BBB has minimal regenerative capacity at the endothelial layer; the syncytiotrophoblast is replaced continuously through cytotrophoblast fusion, but this fusion is itself the target of NP-induced PERK signalling, a mechanistic feedback loop in which NP exposure simultaneously injures and impairs the repair of the placental barrier.

### 6.4. Comparative Vulnerability Summary

The three barriers differ in terms of the balance between permeability and repair capacity (Table 7). Table 8 provides a parallel summary of evidence strength across the three barriers using the detection–association–mechanism–causality schema. The BBB is the most receptor-mediated transcytosis-permissive but has the lowest repair capacity; the placenta is the most actively bidirectional and has its repair machinery directly targeted; the intestine is the most paracellular-leakage-prone under inflammatory conditions but is rapidly renewed.

Key mechanistic insight (Section 6): Predictions of NP risk must be tissue-stratified rather than aggregated under a generic “biological barrier” label; the molecular heterogeneity of junctional architecture, transporter expression, ROS sensitivity, and repair capacity across the three barriers provides the mechanistic rationale for that stratification.

Knowledge gap (Section 6): Comparative quantitative permeability data for identical, environmentally relevant NPs in matched human-derived intestinal, BBB, and placental microphysiological systems do not yet exist.

## 7. Cellular and Molecular Toxicity Mechanisms

### 7.1. Hierarchical Oxidative Stress and the Nrf2–Keap1 Axis

Across cell types studied in vitro and in vivo in animals, ROS generation is consistently reported as an early molecular event of NP exposure. The hierarchical oxidative-stress paradigm—Tier 1: Nrf2 activation, HO-1 induction, GSH synthesis; Tier 2: redox-sensitive MAPK/NF-κB-driven inflammation; Tier 3: mitochondrial collapse and apoptosis originally formulated for combustion-derived ultrafine particles, maps coherently onto the NP literature [65,88].

At Tier 1, the Keap1–Nrf2 axis is the principal cytoprotective sensor: under basal conditions, Keap1 targets Nrf2 for proteasomal degradation, but oxidation of redox-active cysteines on Keap1 releases Nrf2 to transactivate antioxidant-response-element genes including HO-1, NQO1, GCLC/GCLM and thioredoxin reductase [116,117]. Nrf2 directly antagonises NF-κB signalling, providing the molecular basis for the Tier 1 → Tier 2 switch when sustained ROS exceeds antioxidant capacity. The conserved nature of this cascade is consistent with NP-driven pathology behaving as a generic redox stressor rather than a polymer-specific phenomenon, which would simplify risk assessment but argues against biomarker approaches that seek polymer-specific molecular signatures.

### 7.2. Mitochondrial Dysfunction and Quality Control

PS NPs depolarise mitochondrial membranes, reduce ATP synthesis, increase mitochondrial ROS, and trigger Bax translocation with cytochrome c release in HepG2, Caco-2, BMVEC, and trophoblast models in vitro [34]. Mitochondrial dysfunction has also been implicated in glycolipid-metabolism disturbances induced by oral PS-NP administration in mice, mediated through ROS → Nrf2 and NF-κB → MAPK cross-talk [118].

Damaged mitochondria are normally cleared by selective autophagy (mitophagy) via the PINK1–Parkin and BNIP3/NIX pathways [119,120]. When mitophagic flux is overwhelmed, damaged mitochondria persist, and mitochondrial DNA leakage into the cytosol can engage the cGAS-STING innate-immunity pathway—a candidate route by which sustained low-level NP exposure could prime chronic inflammation without producing overt cell death. Direct evidence for mitophagy dysregulation by nanoplastics is currently limited.

### 7.3. Inflammasome Activation, Pyroptosis, and ER-Stress Cross-Talk

NLRP3-inflammasome assembly downstream of ROS produces IL-1β and IL-18, with caspase-1-dependent gasdermin-D cleavage triggering pyroptosis. This pathway is prominent in PS-MP-induced intestinal-barrier dysfunction [27] and is a candidate mechanism by which NPs prime systemic low-grade inflammation—the inflammatory milieu implicated in cardiovascular disease, where Marfella et al. [14] reported elevated inflammation markers in MNP-positive carotid plaques.

ER stress and inflammasome activation do not act as independent pathways; the two engage in crosstalk: *ATF4* and CHOP upregulate TXNIP, a key NLRP3 priming factor; ER-stress-driven Ca^2+^ release into the cytosol potentiates NLRP3 oligomerisation; and IRE1α (a parallel UPR sensor) signals to TRAF2 and JNK to amplify pro-inflammatory transcription [121]. This crosstalk provides a candidate molecular link between the placental PERK/eIF2α/*ATF4* pathology described in Section 5.3 and the systemic NLRP3-driven inflammation observed in NP-exposed animal models.

### 7.4. Genotoxicity and Genome Instability

Reports of direct genotoxicity (single- and double-strand breaks and micronuclei) are inconsistent across studies and depend on cell type, exposure duration, and particle dose. In primary human peripheral-blood lymphocytes, Berber et al. [59] reported reduced mitotic index and dose-dependent increases in micronucleus frequency and comet-assay parameters over 0.001–100 µg/mL of 50 nm PS NPs. This pattern sits between the Liang et al. [27] report of overt genotoxic stress at high mouse doses and the negative findings of Domenech et al. [26], in which 30 nm PS NPs administered to differentiated human Caco-2 monolayers for 8 weeks produced minimal genotoxic signal. A complementary Caco-2 study reported that PS NPs do not directly cause SSBs/DSBs (γH2AX and comet assays both negative) but instead downregulate base excision repair and double-strand-break repair genes, suggesting sublethal genome instability rather than overt genotoxicity [29]. The discordance may reflect cell-type sensitivity (proliferating circulating lymphocytes versus differentiated intestinal-epithelial monolayers), corona composition, and dose range. The non-mutagenic but DNA-repair-suppressing pattern has regulatory implications: such an agent would fail standard genotoxicity assays yet may amplify the impact of co-exposure to genotoxic pollutants, supporting a dedicated mechanism-of-action category in NP risk assessment.

### 7.5. Epigenetic Regulation

NPs and MPs alter DNA-methylation patterns (global hypermethylation following PE-MP exposure; gene-specific changes via DNMT1/DNMT3A upregulation), histone modifications, and non-coding RNA expression, including extracellular-vesicle-borne miRNAs [30,112]. Of particular interest is transgenerational epigenetic inheritance—gestational maternal NP exposure has been linked to altered methylomes and phenotypes in F1 and F2 offspring in mice [111]. This area is methodologically challenging but extends the relevant toxicological question from acute exposure to a developmental-origins-of-disease framework.

### 7.6. Autophagy–Lysosome Dysfunction

A recurring observation in in vitro NP studies, regardless of cell type, is intracellular accumulation of particles within Rab7^+^/LAMP2^+^ lysosomal compartments (Section 3.1). Ragusa et al. [92] reported lysosomal destabilisation, autophagic blockade, and ER- and mitochondria-coupled cytoplasmic alterations in cultured human trophoblasts exposed to PS NPs. The molecular mechanism is consistent with lysosomal membrane permeabilisation (LMP), in which accumulated non-degradable particles disrupt lysosomal membrane integrity, release cathepsins into the cytosol, and trigger inflammasome activation by NLRP3—closing a feed-forward loop between Section 7.3 and Section 7.6 [122]. LMP also impairs the resolution of autophagic flux: autophagosomes form normally but cannot complete fusion with permeabilised lysosomes, producing an accumulation of damaged organelles and protein aggregates that further amplifies cellular stress. The convergence of NLRP3 inflammasome activation, ER stress, mitochondrial damage, and lysosomal compromise on chronically NP-exposed cells therefore plausibly accounts for the persistent low-grade cellular dysfunction observed even at sub-cytotoxic doses.

### 7.7. Protein Aggregation and Templated Misfolding

Anionic PS NPs nucleate α-synuclein fibrillation with high specificity to the NAC region [36]. In parallel, sub-diffraction infrared imaging has shown that PS surfaces template β-sheet-rich amyloid-β conformations in neuronal cells, supporting a role for PS as a catalytic scaffold for amyloidogenic misfolding [37]. By analogy with other amyloidogenic proteins, NP surfaces could plausibly accelerate amyloid-β and tau aggregation, providing a candidate molecular bridge between environmental exposure and neurodegenerative disease that does not require the NP itself to be neurotoxic. This remains a hypothesis grounded in biophysical in vitro assays only; it offers one candidate mechanistic framework for the dementia–MNP correlation reported by Nihart et al. [18], but requires validation in cellular and animal models before being treated as established.

Key mechanistic insight (Section 7): Reports across cell types and species are consistent with a tissue-conserved redox cascade and tissue-specific Tier-2 inflammatory amplification, complicated by sublethal genome instability, epigenetic remodelling, mitochondrial quality-control failure, autophagy–lysosome dysfunction, ER stress/inflammasome cross-talk, and more speculatively templated protein misfolding. These downstream effects are independent of the upstream barrier-translocation question and may be relevant whether intact particles cross the relevant barrier or not.

Knowledge gap (Section 7): Quantitative dose–response data linking realistic human exposure (µg L^−1^ to ng L^−1^ in plasma) to molecular biomarker thresholds (oxidative stress, ER stress, methylation) are largely absent.

## 8. Methodological Limitations and Sources of Uncertainty

Any synthesis of NP toxicology has to engage with the methodological fragility of the underlying evidence, and we expand that discussion here because the credibility of conclusions about human nanoplastic translocation rests on it.

### 8.1. Detection and Quantification: False Positives and False Negatives

The two dominant techniques are vibrational spectroscopy (µ-FTIR and Raman) and pyrolysis-GC/MS. µ-FTIR has a practical lower size limit of ~10–20 µm; µ-Raman extends this to ~1 µm; Py-GC/MS quantifies polymer mass without size or count information and destroys the sample [123]. No current routine technique reliably quantifies sub-micrometre NPs in biological tissues [3,4]. Reported concentrations in human samples therefore systematically underestimate particle counts in the size range most likely to cross biological barriers, while concurrently risking false positives from polymer-matrix interference and laboratory contamination.

The risk of analytical false positives is method-specific. In Py-GC/MS, several characteristic pyrolysis markers used for polymer identification overlap chemically with natural-product pyrolysates abundant in lipid- and protein-rich tissues, raising the possibility that a biogenic signal is misclassified as a polymer in matrices such as brain or placenta [3,4]. In µ-Raman and µ-FTIR, pigment particles can produce spectra that are misclassified as polymers by automated library-matching algorithms, an issue acutely illustrated by Ragusa et al. [8], in which only 3 of 12 detected microparticles could be unambiguously assigned to a polymer (Section 5.4). False negatives dominate at the small-particle end: any NP below the spatial resolution of the detection technique is missed, biasing apparent size distributions toward larger particles. Recovery efficiency during tissue digestion is rarely characterised on spiked tissues, and partial polymer degradation during alkaline digestion can shift detectable particles below detection limits [13,124]. Quantitative human-tissue claims should be accompanied by explicit reporting of recovery efficiency on matrix-matched reference materials.

Several emerging analytical modalities are beginning to close the sub-micrometre detection gap and warrant explicit mention. Optical photothermal infrared spectroscopy (O-PTIR) couples a mid-infrared pump laser to a visible probe and achieves a lateral resolution near 400–500 nm that is decoupled from the infrared diffraction limit, enabling polymer-specific chemical mapping of individual sub-micrometre particles in biological matrices without the spatial constraints of conventional µ-FTIR. Atomic force microscopy-infrared spectroscopy (AFM-IR) and scattering-type scanning near-field optical microscopy (s-SNOM) extend chemical fingerprinting to the tens-of-nanometres scale through tip-based detection, and tip-enhanced Raman spectroscopy (TERS) provides analogous nanoscale Raman contrast. Stimulated Raman scattering (SRS) microscopy offers label-free, polymer-selective imaging at video rate and has been applied to particulate contaminants in tissue sections. In parallel, single-particle inductively coupled plasma mass spectrometry (sp-ICP-MS) is being adapted for sub-micrometre polymer particle counting, either via intrinsic heteroatoms or via metal-tagged reference materials. These techniques carry their own caveats: limited sample throughput, contamination risk during nanoscale tip approach, high instrumentation cost, restricted inter-laboratory standardisation, and, for tip-based methods, sample preparation requirements that themselves introduce plastic-contact steps. Systematic benchmarking against Py-GC/MS on matrix-matched reference materials will be required before quantitative human-tissue claims at the true nanoscale can be supported [125,126,127,128,129].

### 8.2. Contamination Control and Endotoxin Confounding

Plastic is ubiquitous in laboratory environments—sample tubes, pipette tips, filter housings, lab-coat fibres, ambient air—and contamination at every stage of sample collection, processing, and analysis is a real and persistent risk [5,6]. A rigorous inter-laboratory study found procedural blanks containing 7–511 microparticles per sample, with a mean of 80 ± 134 [130]; ~20% of published microplastic studies report no procedural blanks [131]. For low-µg-per-gram tissue concentrations, contamination control is among the most consequential methodological choices, and quantitative human-tissue claims should be accompanied by field and procedural blanks and explicit blank-subtraction protocols.

A complementary contamination problem affects in vitro and in vivo toxicology: bacterial endotoxin (LPS) contamination of commercial nanoparticle preparations is widespread and under-recognised. Owing to their high surface area, nanoparticles readily adsorb LPS from non-sterile handling, and even ng-per-mL contamination produces inflammatory cytokine release indistinguishable from genuine nanoparticle-driven inflammation. Standard LAL detection is itself susceptible to nanoparticle interference, generating false-negative LPS measurements [132,133]. Inflammatory readings from NLRP3/inflammasome studies (Section 7.3 and Section 7.6) that did not include endotoxin controls validated by interference-corrected assays should be interpreted with caution.

### 8.3. Dose Realism and the Translational Gap

Most in vivo and in vitro NP toxicology uses doses that exceed plausible human exposure by 3–6 orders of magnitude. Cox et al. [54] estimated American annual dietary microplastic intake at 39,000–52,000 particles per person (74,000–113,000 with inhalation); at a conservative ~1 ng per particle this corresponds to ng-to-µg per day. Animal in vivo studies routinely use 0.1–10 mg L^−1^ in drinking water [28], or 0.5–50 mg kg^−1^ body weight by gavage [32], and in vitro studies typically use 10–500 µg mL^−1^—four to six orders of magnitude above body-weight-normalised plausible human exposure. A small number of studies have explored lower ranges: Berber et al. [59], for example, tested 0.001–100 µg/mL in human lymphocytes and reported cytogenotoxicity at the lower end of this range, although the ex vivo whole-blood exposure design (24–48 h) differs from chronic human exposure. Many of the reported toxic effects may therefore not reflect realistic human exposure, and this caveat warrants explicit treatment in every primary study. Simple dose scaling is insufficient because corona composition, half-life, and tissue distribution change non-linearly with concentration.

### 8.4. Labelling Artefacts and Dye Leakage

Most in vitro and in vivo uptake studies employ commercial fluorophore-labelled PS particles. Nile red and BODIPY leaching from the polymer matrix into surrounding lipids has been documented repeatedly [89,90]; a signal apparently indicating intracellular NP localisation may therefore reflect free dye that has escaped its carrier and partitioned into lipid droplets, membranes, or lipid-rich tissue regions. The artefact disproportionately affects long-exposure studies and lipid-rich compartments—exactly those of greatest interest at the BBB, placenta, and liver—and is particularly problematic for high-impact studies that claim NP entry on the basis of fluorescent colocalisation alone without orthogonal label-free confirmation. Robust quantification requires label-free methods such as Py-GC/MS, hyperspectral imaging, or covalently labelled tracers [21].

### 8.5. Particle Realism, Aggregation, and DLS Limitations

Nearly all toxicological data come from monodisperse, commercial, surfactant-stabilised PS spheres. Environmentally weathered NPs are heterogeneous in size and shape, oxidised, additive-leaching, and polymer-diverse [48]; dose–response relationships from pristine-PS studies should therefore be treated as upper-bound benchmarks of biological reactivity rather than realistic exposure simulations.

Even within the pristine-PS literature, the particle population presented to cells is rarely what the manufacturer’s label states. On dilution into protein-containing culture medium, surfactant-stabilised PS NPs are partially decoated by protein corona and undergo rapid aggregation into clusters spanning hundreds of nanometres to micrometres [20,129], yet most studies report the nominal diameter as if biologically relevant. Dynamic light scattering—the most widely used sizing method—returns an intensity-weighted average biased toward the largest scatterers and cannot reliably distinguish primary particles from agglomerates in polydisperse or protein-decorated suspensions [134]. Particle size should be characterised in the exposure medium at the exposure time-point with complementary methods such as nanoparticle tracking analysis, differential centrifugal sedimentation, or electron microscopy, but this is rarely done.

### 8.6. Protein Corona Misinterpretation and Serum-Source Dependence

Coronae are typically pre-formed in foetal bovine serum, a non-physiological heterologous matrix, and characterised by mass spectrometry of the hard fraction only; the soft corona, which dominates the dynamic biological identity, is rarely quantified. Coronae formed in human plasma differ in composition and lipid content from FBS-formed coronae [21], and human-plasma coronae themselves vary by donor, by storage condition, and by post-prandial state. Corona evolution at the gut lumen, CSF, or maternal–foetal interface is essentially uncharacterised. Mechanistic claims that hinge on corona-mediated receptor engagement therefore require fluid-matched, time-resolved corona characterisation that few published studies currently provide.

### 8.7. Batch-to-Batch Variability of Commercial Particles

Even nominally identical commercial PS NPs from the same supplier vary between production batches in mean diameter, size-distribution width, residual surfactant, surface-charge density, and trace endotoxin content. This variability is rarely controlled for and contributes to inter-laboratory disagreement on basic outcomes such as Caco-2 cytotoxicity thresholds and BBB transwell permeability. Robust studies should characterise the specific particle lot used and report at minimum the hydrodynamic diameter in the exposure medium, zeta potential, polydispersity index, surfactant identity and residual concentration, and an endotoxin measurement performed with an interference-controlled assay.

### 8.8. Lack of Standardisation and Cross-Study Reproducibility

Reported NP loads in identical biological matrices vary by 1–3 orders of magnitude across laboratories [13], and no internationally agreed reference materials, contamination-control protocols, sample-preparation procedures, or detection limits exist. This heterogeneity remains a major obstacle to translating the NP-detection literature into actionable risk estimates. Inter-laboratory round-robin standardisation exercises across human-relevant matrices, with both pristine and weathered reference NPs, are overdue.

Key mechanistic insight (Section 8): The methodological deficiencies of the field substantially constrain the interpretation of mechanistic claims. Meaningful progress will require improvements in particle realism, label-free orthogonal quantification, fluid-matched corona analysis, controlled endotoxin assessment, batch-level particle characterisation, and rigorous contamination controls, without which prominent human-tissue findings cannot be reliably interpreted.

Knowledge gap (Section 8): A coordinated international round-robin reference programme using both pristine and weathered NPs across human-relevant matrices is overdue; without it, future human-tissue concentration claims will continue to vary inconsistently between laboratories.

### 8.9. Consolidated Sources of Bias in NP Barrier Studies

Six sources of bias run through the sections above and jointly condition every mechanistic claim reviewed in this manuscript. First, particle realism: Pristine, monodisperse, surfactant-stabilised PS beads dominate the empirical record, and their behaviour is a poor proxy for weathered, additive-loaded environmental NPs. Second, dose realism: Experimental exposures typically exceed plausible human intake by 3–6 orders of magnitude, so dose–response extrapolation to human relevance is not straightforward. Third, detection realism: No current routine method reliably quantifies sub-micrometre polymer particles in biological tissue, so tissue burdens are systematically underestimated at the small-particle end while remaining vulnerable to library-matching false positives at the resolvable end. Fourth, corona realism: coronae are still frequently formed in foetal bovine serum rather than in compartment-matched human fluids. Fifth, single-centre and single-laboratory dominance: several of the most-cited human findings (plaque hazard ratio [14], brain accumulation [18], PERK-axis trophoblast pathology [42]) rest on individual laboratories or centres and have not been independently replicated under stringent contamination control. Sixth, publication and citation bias: positive and mechanistically striking findings are systematically more likely to appear in high-visibility outlets than replication attempts or null results. Any interpretation of the mechanistic literature reviewed here has to be read against this composite bias structure. Table 9 lists selected controversial or contradictory findings across the three barriers, with candidate methodological reasons.

## 9. Future Directions

### 9.1. Microphysiological Systems and the CDBS Test

A direct test of the CDBS hypothesis would require head-to-head NP translocation studies using identical, environmentally weathered reference NPs across matched human-derived BBB-on-chip, gut-on-chip, and placenta-on-chip systems, with corona pre-equilibration in compartment-matched fluids and label-free Py-GC/MS readouts. Such platforms now exist with sufficient physiological fidelity: hypoxia-induced iPSC-derived BMVEC chips reproduce LRP1- and TfR-mediated transcytosis [79,80,135]; placenta-on-chip systems with co-cultured BeWo trophoblasts and HUVECs under shear stress recapitulate syncytialisation dynamics and selective transfer [136]; intestine-on-chip platforms incorporate mucus, microbiota, and immune cells [137].

A minimum viable experimental matrix to test CDBS would combine the following axes deployed factorially in the three matched microphysiological systems above.

Particle axis: (a) Pristine PS spheres of 50, 200, and 500 nm as reference materials; (b) UV/ozone- and shear-weathered PS, PE, and PET fragments of matched hydrodynamic diameter and comparable ζ-potential; (c) at least one additive-loaded weathered fragment (for example, phthalate-leaching PVC) to separate polymer-intrinsic from leachate-driven effects.

Corona pre-equilibration axis: Identical particles pre-incubated in (i) simulated human chyme, (ii) human plasma, and (iii) intervillous-space-mimicking fluid, with proteomic and lipidomic characterisation of the resulting coronae before exposure to the corresponding barrier chip.

Dose axis: At least three log-spaced concentrations bracketing the highest plausible human exposure estimate (µg L^−1^ range) rather than the mg L^−1^ regimes typical of the current literature, together with a positive control at conventional experimental doses to allow cross-comparison with published work.

Read-out axis: Label-free particle translocation quantified by Py-GC/MS or sp-ICP-MS on the abluminal compartment; barrier integrity by transepithelial/transendothelial electrical resistance and paracellular tracer permeability; corona-receptor engagement by antibody-mediated blockade (anti-TfR1, anti-LRP1, anti-FcRn) and by CRISPR knock-out of candidate receptors in the chip cell lines. Downstream mechanistic read-outs include tight-junction protein imaging, ROS and mitochondrial-membrane-potential probes, and transcriptomic sampling of the barrier cells.

Falsification criterion: CDBS predicts that identical particles will translocate the three barriers at significantly different efficiencies once matched-fluid coronae are formed, and that receptor blockade will selectively abolish translocation in the barrier whose corona-receptor pairing dominates. Failure to observe barrier-specific corona–receptor coupling under these controlled conditions, or observation of equivalent translocation independent of corona composition and receptor availability, would falsify the framework as currently articulated. The matrix is proposed as a road map, not a claim of feasibility in a single laboratory: it defines the minimum controlled comparisons required to move CDBS from hypothesis to tested framework.

### 9.2. Organoids and Stem-Cell-Derived Models

Brain organoids derived from human iPSCs recapitulate cortical layering, neurogenesis, and electrophysiological function [138]. Hua et al. [138] reported that MP exposure to forebrain organoids transiently promoted neural-progenitor proliferation but, with extended exposure, downregulated mature neuronal markers and cortical layer VI markers—a phenotype consistent with subtle developmental neurotoxicity that would be invisible in adult monocultures. Trophoblast organoids [139] and intestinal organoids offer analogous opportunities for the placental and gut barriers.

### 9.3. Multi-Omics Integration

Single-cell RNA-seq, spatial transcriptomics, methylome analysis, and proteomics applied to NP-exposed barriers will move the field beyond NF-κB activation to high-resolution mechanistic maps. Microglial heterogeneity post-NP exposure is a particularly fruitful target.

### 9.4. Molecular Biomarkers for Human Cohort Studies

Translatable biomarkers are essential for human studies. Promising candidates include plasma S100B and neurofilament-light-chain for BBB integrity; zonulin and intestinal fatty-acid binding protein for gut barrier; placental growth factor and sFlt-1 for placental dysfunction; and miRNA panels in extracellular vesicles for systemic inflammatory tone. Coupling these with quantitative Py-GC/MS NP measurements in matched plasma, urine, and stool samples—with rigorous procedural and field blanks—could enable epidemiological analyses with mechanistic anchoring.

### 9.5. Long-Term and Transgenerational Studies

Almost all in vivo NP toxicology uses exposures of weeks; Wang et al. [34] reported one of the few examples of latent post-exposure pathology, showing neuronal damage 10 months after a 1-week exposure. Cross-generational studies are still rarer. Given that human NP exposure is lifetime and begins in utero, the temporal mismatch is one of the most consequential gaps in current evidence. Lifelong NP exposure cohorts in mice and well-designed prospective birth-cohort studies in humans are needed.

### 9.6. Reference Materials and Inter-Laboratory Harmonisation

International weathered-NP reference materials, contamination-control protocols, and inter-laboratory round-robin quantification will be foundational. Without these, the field will continue to generate divergent estimates that cannot inform regulation under REACH, EFSA, or FDA frameworks.

Key mechanistic insight (Section 9): The next decade of NP research should shift from new exposure documentation toward mechanistic depth, methodological standardisation, and integrative human cohort science.

Knowledge gap (Section 9): No funding agency has yet implemented a coordinated, multi-barrier, multi-omic, longitudinal human NP exposure programme—a structural deficiency that limits the translational power of the field.

### 9.7. Prioritised Research Agenda

Consolidating the section-by-section suggestions above, we identify three to four highest-impact directions per barrier, together with a near-term feasibility indicator.

Intestinal barrier: (i) Integrated mucus–microbiota–epithelial–immune co-culture platforms exposed at µg L-1 doses (feasible now, existing chip and organoid systems mature); (ii) longitudinal human dietary-intake studies with paired stool Py-GC/MS and epithelial biomarkers (feasible medium-term, requires cohort infrastructure); (iii) mechanistic disentanglement of leachate-driven versus polymer-driven epithelial injury using additive-stripped weathered particles (feasible now); (iv) systematic testing of the IBD-MP correlation with prospective, not cross-sectional, designs (feasible medium-term).

Blood–brain barrier: (i) Label-free particle quantification (Py-GC/MS or sp-ICP-MS) on iPSC-BBB and brain-chip effluents to remove fluorescence artefacts (feasible now); (ii) targeted receptor-blockade and CRISPR knock-out of TfR1, LRP1, and FcRn in iPSC-BMVECs to test corona–receptor coupling (feasible now); (iii) independent replication of Nihart et al.’s brain-accumulation dataset with orthogonal analytical methods (feasible short-term, requires autopsy access); (iv) longitudinal cohorts pairing plasma NP burden with cognitive and neuroimaging endpoints (feasible long-term).

Placental barrier: (i) Placenta-on-chip exposure to matched-fluid coronae at physiological shear stress with label-free translocation readouts (feasible now); (ii) systematic quantification of bidirectional transfer as a function of particle size, polymer type and additive loading in matched ex vivo perfusion (feasible now); (iii) prospective birth-cohort studies with maternal-foetal biomonitoring paired to pregnancy outcomes (feasible medium-term); (iv) mechanistic testing of the syncytialisation-failure phenotype in primary human trophoblasts across independent laboratories to establish reproducibility (feasible short-term).

Cross-cutting: Standardisation of weathered reference materials, contamination-control protocols, and analytical benchmarking is a prerequisite for any of the above to yield comparable data across laboratories (feasibility depends on coordinated funder and regulator action).

### 9.8. Regulatory and Public Health Implications

Improved mechanistic understanding of nanoplastic barrier crossing is directly relevant to risk assessment and to policy in three respects. First, it should refine reference-dose thinking current toxicology relies on doses far above human exposure and cannot be transferred directly into regulatory margins of exposure without dose-realistic re-testing. Second, it should inform prioritisation of polymer types and additives for regulatory attention: additive leachates rather than the polymer backbone may dominate the toxicological signature of weathered fragments, which has implications for chemical rather than particle regulation. Third, it should shape public communication: the current evidence supports concern and continued investment but does not support quantitative causal statements about specific human diseases, and messaging that outruns the evidence risks eroding public trust in the field. Explicit, evidence-level-stratified communication to policymakers and the public is therefore itself part of the research agenda, not an addendum to it.

## 10. Conclusions

Polymer signatures of nanoplastics have been detected in placenta, blood, lung, atherosclerotic plaque, testis, semen, olfactory bulb, and brain. The open mechanistic questions concern how much of the apparent signal is genuine, by what biomolecular pathways NPs cross specific barriers, and with what tissue-specific consequences. This review has argued that the three most clinically important barriers—intestinal, blood–brain, and placental—are not interchangeable filters but molecularly distinct interfaces. As one organising lens for further work, we have outlined a corona-driven barrier selectivity (CDBS) hypothesis, in which each barrier samples incoming NPs through a tissue-specific corona that engages a tissue-specific receptor repertoire; this hypothesis remains experimentally untested.

A common mechanistic skeleton emerges from animal and in vitro data: ROS generation initiates an NF-κB- and (in some compartments) NLRP3-driven inflammatory cascade; ER stress engages the PERK/eIF2α/*ATF4* axis with particularly clear consequences at the placental syncytiotrophoblast; tight-junction proteins are downregulated secondarily, allowing paracellular leakage; and downstream sublethal genome instability and epigenetic remodelling extend toxicity beyond the directly exposed cell.

However, we underline the limits of the evidence base. The dominant pristine PS-bead model is poorly representative of weathered, additive-leaching environmental NPs. Detection methods underestimate sub-micrometre particles and risk false positives from contamination and matrix interference. Coronae are studied in foetal bovine serum rather than in compartment-matched human fluids. Inter-laboratory variability is large. Experimental doses are commonly 3–6 orders of magnitude above realistic human exposure. The Marfella et al. [14] cardiovascular hazard ratio, the Nihart et al. [18] brain accumulation, and the Cheng et al. [42] PERK-axis trophoblast pathology are among the most-cited findings in this area, but each remains a single-centre or single-laboratory report awaiting independent replication under stringent contamination control.

Causal links between NPs and inflammatory bowel disease, neurodegeneration, atherosclerosis, or adverse pregnancy outcomes remain hypothesis-generating rather than established. Priorities for the next phase of the field include (i) standardised weathered reference materials and contamination protocols; (ii) experimental testing of corona-driven hypotheses such as CDBS in matched human microphysiological systems; (iii) longitudinal human cohort science with rigorous Py-GC/MS quantification and mechanistic biomarkers; and (iv) explicit, evidence-level-stratified communication of findings to policymakers. The biomolecular mechanisms reviewed here are coherent enough, and the human evidence suggestive enough, to justify continued public-health investment in this area; at the same time, the methodological constraints remain severe enough that strong causal claims should still be avoided.

## Figures and Tables

**Table 1:** Consolidated hierarchy of human evidence for micro- and nanoplastic detection across tissues, with analytical method, evidence level and evidence category. Categories follow the detection–association–mechanism–causality schema used throughout the review. Reference numbers refer to the manuscript reference list.

Tissue/Matrix	Representative Study (as Cited)	Analytical Method	Evidence Level	Evidence Category	Key Caveat
Stool	Ref. [7]	µ-Raman/µ-FTIR	Human in vivo	Detection	Reflects transit rather than absorbed dose; sub-µm particles below detection limit
Placenta	Ragusa et al. [8]; Garcia et al. [9]	µ-Raman; Py-GC/MS	Ex vivo human	Detection (partial); only 3/12 particles unambiguously assigned as polymer in [8]	Contamination during obstetric handling; polymer misassignment risk on library-matching
Lung tissue	Refs. [10,11]	µ-Raman/µ-FTIR	Ex vivo human	Detection	Sub-µm particles undercounted; airborne contamination during resection
Whole blood	Refs. [12,13]	Py-GC/MS	Human in vivo	Detection	Small cohorts; blank correction dominates apparent signal in some laboratories
Atherosclerotic plaque	Marfella et al. [14]	Electron microscopy + spectroscopy	Human in vivo (single-centre prospective)	Association (HR 4.53 for MI/stroke/death over 34 months)	Single centre, no independent replication; residual confounding acknowledged
Olfactory bulb	Ref. [15]	µ-Raman/µ-FTIR	Ex vivo human	Detection	Small autopsy cohort; nasal-cavity contamination pathway not excluded
Testis and semen	Refs. [16,17]	Vibrational spectroscopy/Py-GC/MS	Ex vivo human/ human in vivo	Detection	Sample-handling contamination; polymer assignment uncertainty
Frontal cortex (brain)	Nihart et al. [18]	Py-GC/MS + ATR-FTIR + EM	Ex vivo human (decedent)	Detection (contested magnitude); association with dementia diagnosis	Lipid co-pyrolysis interference [3,4]; cross-sectional; reverse causation plausible

**Table 2:** Barrier-specific corona partners and candidate receptor/transport machinery within the corona-driven barrier selectivity (CDBS) framework. Column entries synthesise the discussion in Section 1.1, Section 2.3 and Section 6.2 of the manuscript; evidence levels follow the schema applied throughout the review.

Barrier	Compartment Fluid (Primary Corona Source)	Reported Dominant Corona Components	Candidate Receptor/Transport Machinery	Evidence Level
Intestinal	Chyme, mucus overlay, intestinal interstitial fluid	Mucins, dietary lipids, bile-acid-conjugated proteins, IgA	Clathrin-mediated endocytosis, caveolae, M-cell antigen sampling, paracellular route after TJ disruption	in vitro; animal in vivo
Blood–brain	Plasma/CSF	Apolipoproteins (ApoE, ApoB), immunoglobulins, complement factors, lipoprotein particles	TfR1, LRP1, insulin receptor, caveolae, P-gp/BCRP (efflux)	In silico; in vitro; animal in vivo; contested human
Placental	Maternal plasma, intervillous-space fluid	Placental protein hormones, apolipoproteins, IgG (FcRn substrate), lipoprotein particles	FcRn (IgG transport), caveolae, clathrin, ABC efflux transporters	Ex vivo human; in vitro; animal in vivo

**Table 3:** Summary of key molecular findings on nanoplastic translocation across biological barriers, with explicit evidence-level annotations.

Barrier	Particle (Size, Polymer)	Model System	Evidence Level	Key Molecular Finding	Reference
Intestinal	30 nm PS	Caco-2, 8 wk	In vitro human	Accumulation; minimal genotoxicity; sublethal stress	Domenech et al. [26]
Intestinal	50 nm–5 µm PS	Mice; Caco-2	In vivo animal + in vitro	ROS → NF-κB/NLRP3/MLCK → ZO-1/OCLN/CLDN-1 ↓	Liang et al. [27]
Intestinal	PS NPs, 32 wk	Mice, drinking water	In vivo animal	↑ clathrin and caveolin-1; TJ ↓	Li et al. [28]
Intestinal	~100 nm PS	Caco-2	In vitro human	No SSB/DSB; BER/DSB-repair gene ↓	Kustra et al. [29]
Intestinal	PS NPs	Mice + Caco-2	In vivo animal + in vitro	EV-miRNA reprogramming of host–microbe interaction	Park et al. [30]
Intestinal (human)	Various polymers	50 IBD vs. 52 controls	Human cross-sectional	Faecal MP correlates with IBD activity (no causality)	Yan et al. [31]
BBB	50 nm PS, oral 7 d	Mice; hCMEC/D3	In vivo animal + in vitro	BBB permeability ↑; microglial uptake; ROS, NF-κB, TNF-α	Shan et al. [32]
BBB	0.293 µm PS, oral 2 h	Mice; coarse-grained MD	In vivo animal + in silico	NP brain entry within 2 h; corona-modulated bilayer partitioning	Kopatz et al. [22]
BBB	30–50 nm PS, oral	Mice; primary cells	In vivo animal + in vitro	Cognitive dysfunction; preferential microglial uptake	Kim et al. [33]
BBB	50 + 200 nm PS	Mice, 1 wk + 10 mo follow-up	In vivo animal	Latent synapse loss; M1 microglia/A1 astrocyte	Wang et al. [34]
BBB	Anionic PS NPs	APP/PS1 mice	In vivo animal	Aggravated AD-like pathology	Liu, S. et al. [35]
BBB	PS NPs + α-synuclein/amyloid-β	Biophysical	In vitro biophysical	NAC-region binding; β-sheet stabilisation (HYPOTHESIS)	Liu, L. et al. [36]; da Silva et al. [37]
BBB (human)	All polymers	52 frontal cortex autopsies	Human autopsy	~3300 → 4900 µg/g, 2016 → 2024; 3–5× ↑ in dementia	Nihart et al. [18]
Placental	50–500 nm PS	Ex vivo perfused cotyledon	Ex vivo human	M→F transfer up to 240 nm; 500 nm excluded	Wick et al. [38]
Placental	50–300 nm PS	Ex vivo perfused cotyledon	Ex vivo human	Bidirectional, F→M > M→F; energy-dependent	Grafmueller et al. [39]
Placental	50–300 nm PS	BeWo + HPEC-A2	In vitro human co-culture	Limited translocation at 49–70 nm; size-dependent	Aengenheister et al. [40]
Placental	PS NPs	BeWo, ACH-3P, Jeg-3, JAR	In vitro human	↓ viability/tubule formation; ↑ ROS in HUVEC	Dusza et al. [41]
Placental	PS NPs, gestational	Mice + human trophoblasts	In vivo animal + in vitro	PERK/eIF2α/ATF4 ↑; GCM1/syncytin ↓; embryo resorption ↑	Cheng et al. [42]
Placental (human)	Various	Term placenta, µ-Raman, n = 6	Human cross-sectional	12 microparticles (5–10 µm) in 4/6 placentas; only 3/12 chemically identified as PP	Ragusa et al. [8]
Placental (human)	Various	Term placenta, Py-GC/MS, n = 62	Human cross-sectional	6.5–790 µg/g; PE dominant	Garcia et al. [9]
Cardiovascular (human)	MNPs in plaque	304-pt prospective cohort	Human prospective	HR 4.53 (95% CI 2.00–10.27) for MI/stroke/death	Marfella et al. [14]

Abbreviations: AD, Alzheimer’s disease; BBB, blood–brain barrier; BER, base excision repair; BMVEC, brain microvascular endothelial cell; CLDN, claudin; DSB, double-strand break; EV, extracellular vesicle; F→M, foetal-to-maternal; HR, hazard ratio; HUVEC, human umbilical vein endothelial cell; IBD, inflammatory bowel disease; M→F, maternal-to-foetal; MD, molecular dynamics; MI, myocardial infarction; miRNA, microRNA; MNP, micro- and nanoplastic; MP, microplastic; NP, nanoplastic; OCLN, occludin; PE, polyethylene; PP, polypropylene; PS, polystyrene; ROS, reactive oxygen species; SSB, single-strand break; TJ, tight junction; TNF-α, tumour necrosis factor alpha; ZO-1, zonula occludens-1. ↑, upregulated/increased; ↓, downregulated/decreased. Evidence-level annotations refer to the experimental model providing the primary data: in silico (computational simulation); in vitro (monoculture or co-culture cell systems); animal in vivo (rodent studies); ex vivo human (perfused human tissue); human cross-sectional (observational human studies without temporal sequence); human prospective (longitudinal human cohort). Readers should consult the original publications and the methodological caveats discussed in Section 8.

**Table 4:** Comparison of conventional nanoparticle transport frameworks with the corona-driven barrier selectivity (CDBS) framework proposed in this review.

Feature	Conventional Protein Corona Theory	Single-Barrier Receptor-Mediated Transcytosis (RMT) Model	Corona-Driven Barrier Selectivity (CDBS)
Corona treatment	Single equilibrated state acquired in one fluid (typically serum/plasma)	Not central; corona effects usually not modelled explicitly	Sequential, tissue-stratified property that evolves as particles traverse chemically distinct fluid compartments
Barrier scope	Applied to any single interface, typically for opsonisation, clearance, or receptor engagement	Developed for one selective interface at a time, most often the BBB	Explicitly comparative across intestinal, blood–brain, and placental barriers using matched particles
Receptor pairing	Corona composition inferred; receptor engagement discussed post hoc	Pre-designed ligand (e.g., anti-TfR antibody, angiopep-2) targeting one receptor of the chosen barrier	Corona composition paired to the receptor repertoire actually expressed at each barrier (TfR1, LRP1, FcRn, P-gp/BCRP, caveolae)
Particle type	Engineered nanoparticles with defined surface chemistry	Engineered nanomedicines with designed targeting ligands	Environmental particle populations (weathered, heterogeneous, additive-loaded)
Transport view	Descriptive—corona shapes fate but no explicit selection rule	Passive size cut-off is overcome by ligand-directed transcytosis at one barrier	Selection problem: translocation efficiency depends on the corona-receptor match at each barrier
Testable predictions	Corona composition varies with fluid; membrane damage correlates with corona morphology	Ligand engineering can enhance BBB or tumour targeting	Identical NPs preincubated in matched chyme, plasma, and intervillous-space fluid should show barrier-specific translocation and receptor-blockade sensitivity
Current evidence status	Established for engineered NPs in single fluids	Established for engineered nanomedicines with designed ligands	Hypothesis-stage; the integrated framework has not been experimentally validated for environmental NPs

**Table 5:** Representative intestinal-barrier nanoplastic studies grouped by model system, with particle characteristics, dose, duration, main outcome, and an explicit dose-realism column. “Representative” studies refer to the categories of work summarised in Section 3; individual references are those cited in the manuscript. No new studies were added.

Reference	Particle Type/Material	Size (nm)	Dose/Duration	Main Outcome	Dose Realism vs. Plausible Human Intake
Caco-2 monoculture studies (representative, see Section 3)	Pristine PS spheres	50–500	0.1–1000 µg/mL; 24–72 h acute	TJ protein loss (ZO-1, occludin, claudin-1), ROS, reduced TEER, clathrin/caveolin-1 upregulation	Upper doses exceed plausible human intake by ~3–5 orders of magnitude
Intestine-on-chip/co-culture (representative)	Pristine PS spheres, some carboxylated	50–200	µg/mL range; days	Barrier dysfunction only at higher doses; mucus layer partially attenuates uptake	Mid-range doses closer to but still above plausible human intake
Rodent gavage (representative)	Pristine PS spheres; some PE, PVC	50–1000	0.5–50 mg/kg body weight; days to weeks	TJ downregulation, microbiome shifts, NF-κB/NLRP3 activation, and some multigenerational effects reported	Doses substantially above plausible human intake
Weathered-particle in vitro (limited)	UV/mechanical-weathered PS, PE, PET	Sub-µm to µm	µg-mg/mL	Altered corona composition, additive leachate-driven cytotoxicity	Not yet standardised; doses variable

**Table 6:** Summary of placental bidirectional transfer findings across model systems and particle types. Entries synthesise the studies cited in Section 5 and Table 3. The “weathered/additive-loaded fragments” row is included to make the current data gap explicit and does not report new data.

Model System	Particle Type/Size	Direction Assessed	Reported Finding	Caveat
Ex vivo human perfusion (representative)	PS spheres, ~50–240 nm	Maternal-to-foetal	Size-dependent transfer; smaller particles cross more readily	Short perfusion time relative to gestation; pristine PS only
Ex vivo human perfusion	PS spheres, ~500 nm	Bidirectional	Some particles detected on both sides; transfer efficiency low	Small sample sizes; contamination controls variable
BeWo trophoblast monolayer	Carboxylated PS spheres	Apical-to-basal (maternal-to-foetal analogue)	Concentration- and size-dependent transfer	Monoculture lacks foetal endothelium; syncytialisation is limited
Placenta-on-chip (co-culture)	PS spheres, ~50–100 nm	Maternal-to-foetal (chip flow)	Detectable transfer under shear stress; barrier integrity partially maintained	Emerging platform; few independent replications
Weathered/additive-loaded fragments	Not systematically tested	-	Data gap	Priority for future work

**Table 7:** Comparative molecular vulnerability of intestinal, BBB, and placental barriers.

Feature	Intestinal	BBB	Placental
Cell type	Enterocytes (+ goblet, M, immune cells)	BMVEC + pericytes/astrocytes	Syncytiotrophoblast (+ cytotrophoblast, foetal endothelium)
Junctional architecture	Heterogeneous claudins (1, 3, 4, 7, 15), occludin, ZO-1	Claudin-5-dominant, claudin-3/-12, occludin, ZO-1/-2, JAM-A	No lateral junctions; trans-syncytial nanopores
Approximate baseline TEER	200–400 Ω·cm^2^	>1500 Ω·cm^2^	50–100 Ω·cm^2^
Dominant transport mode	CME, macropinocytosis, paracellular	RMT (TfR, LRP1, insulin R), caveolar transcytosis	CME, CavME, FcRn, macropinocytosis, ABC efflux
Particle size cut-off (where reported)	<500 nm via M cells; <100 nm enterocytes	<300 nm (corona-dependent, in vivo mouse)	<240 nm (Wick); <70 nm in BeWo co-culture
Polarity	Apical (lumen)–basolateral	Luminal–abluminal	Apical (maternal)–basal (foetal)
ROS/Tier 1 sensitivity	Moderate (high antioxidants but high luminal load)	High (low GSH, energy-intensive)	High (PERK/eIF2α/*ATF4* axis active)
Tier 2 inflammation	NF-κB/NLRP3/MLCK/pyroptosis	NF-κB/MAPK/microglial M1	NF-κB/PERK/impaired syncytialisation
Repair capacity	High (3–5 d enterocyte turnover)	Low (minimal endothelial regeneration)	Continuous via cytotrophoblast fusion (NP-impaired)
Key vulnerable molecule	TJ proteins (ZO-1/OCLN/CLDN-1); MLCK	Claudin-5; LRP1/TfR; microglial homeostasis	*GCM1*, syncytin-1/2; PERK/eIF2α/*ATF4*
Strongest disease-relevant association	IBD [31]	Dementia [18]	Adverse pregnancy outcomes [42]
Confidence in human in vivo mechanistic evidence	Low–moderate	Low–moderate	Low
CDBS prediction	Mucin-rich corona → macropinocytosis + TJ leakage	Plasma corona (ApoE/Tf) → RMT via TfR/LRP1	Maternal blood corona (IgG) → FcRn-mediated transfer

Abbreviations: ABC, ATP-binding cassette; BBB, blood–brain barrier; BCRP, breast cancer resistance protein (ABCG2); BMVEC, brain microvascular endothelial cell; CavME, caveolin-mediated endocytosis; CDBS, corona-driven barrier selectivity; CME, clathrin-mediated endocytosis; FcRn, neonatal Fc receptor; GSH, glutathione; JAM-A, junctional adhesion molecule A; LRP1, low-density lipoprotein receptor-related protein 1; MAPK, mitogen-activated protein kinase; NF-κB, nuclear factor kappa B; NLRP3, NOD-like receptor family pyrin domain containing 3; NP, nanoplastic; PERK, PKR-like endoplasmic reticulum kinase; (ABCB1); RMT, receptor-mediated transcytosis; ROS, reactive oxygen species; TEER, trans-epithelial/endothelial electrical resistance; TfR, transferrin receptor; TJ, tight junction; ZO-1, zonula occludens-1; eIF2α, eukaryotic initiation factor 2 alpha; *ATF4*, activating transcription factor 4; *GCM1*, glial cells missing homolog 1. CDBS predictions in the last row are hypothesis-stage and require experimental validation; they are not established mechanisms. TEER values are approximate and vary substantially by tissue source, culture conditions, and measurement technique. Confidence ratings reflect the cumulative weight of published evidence at the time of writing.

**Table 8:** Summary of evidence strength across the three barriers reviewed, applying the detection–association–mechanism–causality schema. “Strong” denotes multiple independent studies in the specified model system with concordant findings; “Moderate” denotes reproducible but limited replication; “Emerging” denotes small numbers of early studies; “Not established” denotes no adequately powered human causal study.

Barrier	Detection in Humans	Association with Disease	Mechanism In Vitro	Mechanism in Animals	Causality in Humans
Intestinal	Indirect (stool only; no direct epithelial-crossing data in vivo humans)	Ecological correlation with IBD; not established	Strong (Caco-2 monocultures, organoids, co-cultures): TJ disruption, ROS, endocytosis	Moderate (rodent gavage): TJ downregulation, microbiome shifts, NF-κB/NLRP3	Not established
Blood–brain barrier	Frontal cortex Py-GC/MS [18]; contested magnitude	Cross-sectional correlation with dementia diagnosis [18]; reverse causation plausible	Emerging (iPSC-BMVEC chips, Transwell BMVECs): claudin-5 loss, RMT candidates	Moderate (rodent inhalation/gavage): brain-region-specific accumulation, glial activation	Not established
Placental	Ex vivo detection in term placenta [8,9]	No robust epidemiological association yet reported	Strong (BeWo, primary trophoblasts, placenta-on-chip): PERK/eIF2a/*ATF4*, syncytialisation failure	Moderate (murine dams): foetal-tissue detection, growth-restriction phenotypes	Not established

**Table 9:** Selected controversial or contradictory findings across the three barriers, with candidate reasons drawn from the methodological limitations discussed in Section 8.

Barrier	Controversial Finding	Alternative Explanation/Candidate Reason	Reference/Model
BBB	Fluorescent PS beads penetrate intact rodent brain in high amounts after acute exposure	Fluorophore leakage from beads into lipid-rich brain tissue; polymer itself may not be present at the reported level	Rodent in vivo (multiple studies)
BBB	Nihart et al. [18] Py-GC/MS brain MNP concentrations	Lipid co-pyrolysis and styrene-mimetic peptide pyrolysates may inflate PE/PS signal in lipid-rich tissue [3,4]	Ex vivo human decedent brain
Cardiovascular	Marfella et al. [14] HR 4.53 for MI/stroke/death associated with plaque MNP detection	Single-centre prospective study; residual confounding; no independent replication yet	Human in vivo (single centre)
Placental	Only 3 of 12 detected microparticles unambiguously assigned as polymer by Ragusa et al. [8]	Pigment-particle misclassification by automated library-matching; polymer identity uncertain for majority of detections	Ex vivo human placenta
Intestinal	Wide inter-laboratory variability in reported Caco-2 TJ disruption thresholds	Particle surface chemistry, corona formation, serum source, and cell-line drift differ across laboratories; rarely reported to reproducibility standards	Caco-2 monoculture
Cross-barrier	Reported polymer signatures in human blood [12,13] vary by 1–3 orders of magnitude	Blank correction dominates apparent signal in some laboratories; small cohorts; matrix effects	Human whole blood

## Data Availability

No new data were created or analysed in this study. All cited studies are publicly available via the DOIs listed in the reference section.
